# PD-1H/VISTA mediates immune evasion in acute myeloid leukemia

**DOI:** 10.1172/JCI164325

**Published:** 2024-02-01

**Authors:** Tae Kon Kim, Xue Han, Qianni Hu, Esten N. Vandsemb, Carly M. Fielder, Junshik Hong, Kwang Woon Kim, Emily F. Mason, R. Skipper Plowman, Jun Wang, Qi Wang, Jian-Ping Zhang, Ti Badri, Miguel F. Sanmamed, Linghua Zheng, Tianxiang Zhang, Jude Alawa, Sang Won Lee, Amer M. Zeidan, Stephanie Halene, Manoj M. Pillai, Namrata S. Chandhok, Jun Lu, Mina L. Xu, Steven D. Gore, Lieping Chen

**Affiliations:** 1 Division of Hematology/Oncology, Department of Medicine,; 2 Vanderbilt Center for Immunobiology, and; 3 Department of Pathology, Microbiology, and Immunology, Vanderbilt University Medical Center,; 4 Vanderbilt Ingram Cancer Center, Nashville, Tennessee, USA.; 5 Section of Medical Oncology,; 6 Section of Hematology, Department of Medicine, and; 7 Department of Immunobiology, Yale University School of Medicine, New Haven, Connecticut, USA.; 8 Pelotonia Institute for Immuno-Oncology, OSUCCC–James Cancer Hospital,; 9 Department of Microbial Infection and Immunity, The Ohio State University, Columbus, Ohio, USA.; 10 Department of Acute Medicine, Oslo University Hospital, Oslo, Norway.; 11 Department of Internal Medicine, Seoul National University College of Medicine, Seoul, South Korea.; 12 Department of Pathology, New York University Grossman School of Medicine, New York, New York, USA.; 13 Key Laboratory of Digestive System Tumors of Gansu Province, Lanzhou University Second Hospital, Lanzhou, China.; 14 Division of Immunology and Immunotherapy, CIMA, Universidad de Navarra, Pamplona, Spain.; 15 Sylvester Comprehensive Cancer Center, Miller School of Medicine, University of Miami, Miami, Florida, USA.; 16 Department of Genetics and; 17 Department of Pathology, Yale University School of Medicine, New Haven, Connecticut, USA.; 18 National Cancer Institute, Cancer Therapy Evaluation Program, Investigational Drug Branch, Bethesda, Maryland, USA.

**Keywords:** Immunology, Oncology, Cancer immunotherapy, Costimulation, Leukemias

## Abstract

Acute myeloid leukemia (AML) presents a pressing medical need in that it is largely resistant to standard chemotherapy as well as modern therapeutics, such as targeted therapy and immunotherapy, including anti–programmed cell death protein (anti-PD) therapy. We demonstrate that programmed death-1 homolog (PD-1H), an immune coinhibitory molecule, is highly expressed in blasts from the bone marrow of AML patients, while normal myeloid cell subsets and T cells express PD-1H. In studies employing syngeneic and humanized AML mouse models, overexpression of PD-1H promoted the growth of AML cells, mainly by evading T cell–mediated immune responses. Importantly, ablation of AML cell-surface PD-1H by antibody blockade or genetic knockout significantly inhibited AML progression by promoting T cell activity. In addition, the genetic deletion of PD-1H from host normal myeloid cells inhibited AML progression, and the combination of PD-1H blockade with anti-PD therapy conferred a synergistic antileukemia effect. Our findings provide the basis for PD-1H as a potential therapeutic target for treating human AML.

## Abstract 2

<p>Bone marrow blasts from patients with acute myeloid leukemia upregulate PD-1H, which contributes to the induction of immune evasion in murine models.</p>

## Graphical abstract

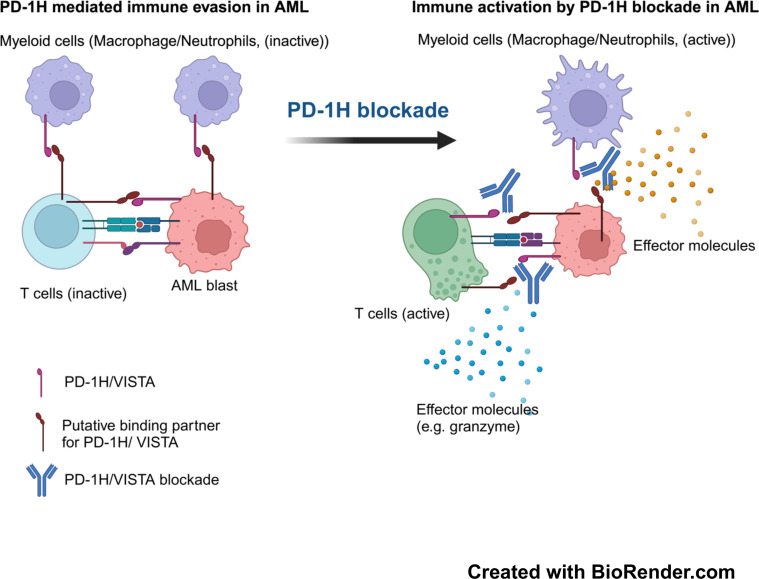

## Introduction

Acute myeloid leukemia (AML) is a heterogenous clonal disorder that is characterized by uncontrolled clonal expansion of myeloid progenitor cells (blasts) that leads to BM failure (1). AML is the most common acute leukemia in adults (1). The incidence of AML is 20,240 diagnoses per year, and over 11,400 patients die annually from this disease in the United States (seer.cancer.gov). Despite progress in our understanding of the pathology and genetics of this disease (2) as well as extensive development of targeted therapeutic modalities (3–15), the mainstay for AML treatment has remained the combination of anthracycline and cytarabine, which was developed in the 1970s (16).

Recently, the antibody therapy targeting the programmed cell death protein 1 (PD-1)/B7-H1 (PD-L1) pathway (collectively called anti-PD therapy) has been at the forefront of cancer therapy (17–20). Anti-PD therapy was developed based on early findings showing selective upregulation of B7-H1 in the tumor microenvironment (TME) by IFN-γ, leading to dysfunction of tumor-infiltrating T lymphocytes (TILs) upon its engagement of PD-1, a mechanism called adaptive immune resistance (18, 19, 21, 22). Currently, anti-PD therapy has been approved by the US FDA for the treatment of more than 25 indications in common cancers, including solid tumors and hematopoietic malignancies (23–36). Despite these exciting developments, clinical efficacy of anti-PD therapy in AML remains obscure. Single-agent anti–PD-1 or anti–PD-L1 mAb trials in AML have shown marginal response rates (37–41). The marginal response to anti-PD therapy in AML indicates that different mechanisms of immune evasion other than the PD pathway may be present.

Programmed death-1 homolog (PD-1H, also known as V domain immunoglobulin suppressor of T cell activation [VISTA], V-set immunoregulatory receptor [VSIR], C10orf54, DD1α, and Gi24) is a coinhibitory molecule of the immunoglobulin superfamily and is broadly found in hematopoietic cells (42, 43). PD-1H delivers an inhibitory signal as a ligand to T cells (43, 44), yet PD-1H on T cells also receives inhibitory signals as a receptor (42, 45–48). Several counterreceptors of PD-1H have been identified, but their immunological functions remain to be elucidated (49–51). PD-1H is expressed mainly in hematopoietic cells, including T cells, monocytes, macrophages, and dendritic cells (42, 44). The presence of PD-1H in normal tissues/cells supports its function as a homeostatic regulator, including maintenance of CD4^+^ T cells in quiescence (45). In preclinical murine models, PD-1H has been shown to induce immune evasion, and genetic ablation or antibody blockade of PD-1H promotes T cell–mediated immunity and suppresses tumor growth (43, 48, 49, 52, 53).

Here, we demonstrate that (a) PD-1H is significantly upregulated in human AML BM, while PD-L1 expression is relatively low; (b) PD-1H is highly expressed on human AML blasts, but not on normal CD34^+^ progenitors; (c) PD-1H expressed on AML blasts contributes to the induction of immune evasion in murine AML models; (d) genetic ablation or antibody blockade of PD-1H reverses immune evasion, leading to antileukemia effects in murine AML models and humanized AML models; and (e) the effect of anti–PD-1H mAbs could be maximized by blocking the PD pathway in murine AML models and humanized AML models.

## Results

### VSIR mRNA is highly upregulated in AML and correlated with poor survival.

We and others have previously reported that PD-1H is broadly expressed on mouse normal hematopoietic cells, including myeloid immune cells and T cells (42–44). PD-1H was also reported to be expressed in some human solid tumor tissues, including prostate cancer (54), pancreatic cancers (55, 56), and melanoma (55, 57, 58), mostly in tumor-infiltrating immune cells. By analyzing the Cancer Genome Atlas (TCGA) database, we found that expression of *VSIR* (PD-1H) mRNA in AML is the highest among over 30 different human cancer types (Supplemental Figure 1A; supplemental material available online with this article; https://doi.org/10.1172/JCI164325S1) (TCGA Research Network, 2013. In addition, *VSIR* is one of the coinhibitory molecules that are expressed at higher levels than others in AML (Supplemental Figure 1B). We next determined *VSIR* expression among AML subgroups based on the French-American-British classification of AML using TCGA. Interestingly, M4 (myelomonocytic) and M5 (monocytic) AML revealed the highest expression of *VSIR* among AML subsets (Supplemental Figure 1C). These findings are consistent with preferential expression of *VSIR* on normal myeloid cells.

We next investigated whether *VSIR* expression is associated with cytogenetic and molecular aberrations that determine the prognosis of AML (59–61). For instance, AML harboring RUNX1-RUNX1T1 (t[8;21]), PML-RARα (t[15;17]), or inv(16) is associated with more favorable prognosis than AML with a complex or monosomal karyotype. *VSIR* expression was significantly lower in favorable-risk AML (i.e., RUNX1-RUNX1T1 [t(8;21)], PML-RARα [t(15;17)]) than in intermediate and poor-risk AML (i.e., intermediate risk: NPM1 mutation, normal karyotype etc.; poor risk: complex karyotype, monosomy [del(5), del(7)], etc.) (Supplemental Figure 1D). However, in some good-risk AML types, such as CBFB-MYH11 (inv[16], t[16;16]), which is often associated with monocytic differentiation, PD-1H showed expression levels comparable to those of intermediate- and poor-risk AML (Supplemental Figure 1D). Although these significant differences in *VSIR* expression were evident based on cytogenetics in AML, molecular mutations including DNMT3A, 11q23 amplification, FLT3, NPM1, and TP53 did not correlate significantly with *VSIR* RNA levels (Supplemental Figure 1E). Therefore, decreased expression of *VSIR* is associated with particular cytogenetic aberrations such as t(8;21) and t(15;17) in AML.

Survival analyses in TCGA to compare the *VSIR*^hi^ quartile AML population with the *VSIR*^lo^ quartile AML population showed that the *VSIR*^lo^ AML population survived longer than the *VSIR*^hi^ AML population (Supplemental Figure 1F). Collectively, our findings suggest a potential role of PD-1H upregulation in immune evasion in AML.

### PD-1H is highly expressed on the surface of human AML blasts.

**Figure 1:**
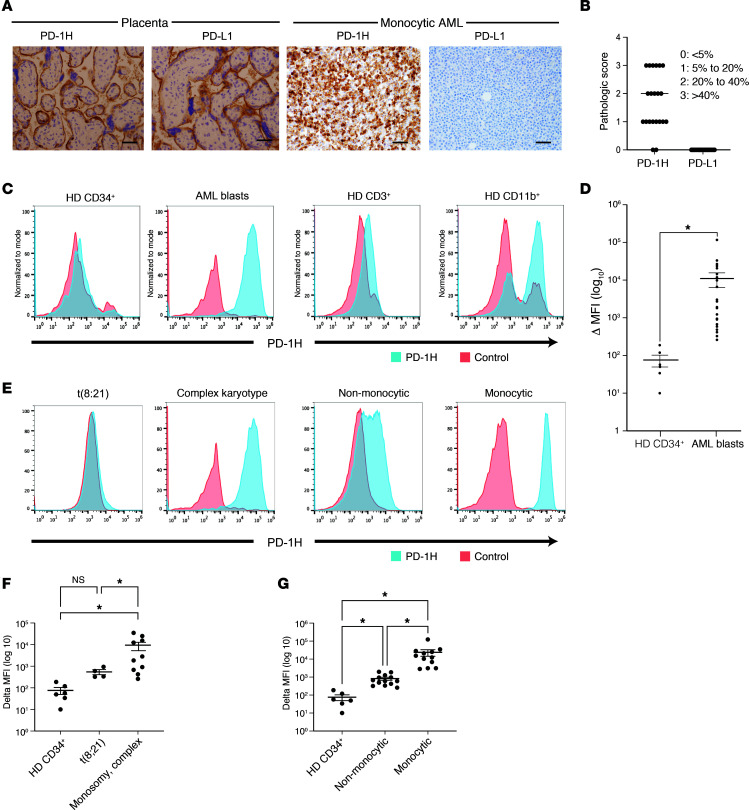
PD-1H protein is highly expressed on AML blasts. (**A**) Immunohistochemical staining of human PD-1H and PD-L1 in AML. Validation of PD-1H and PD-L1 staining in human placenta (left panels). IHC staining of PD-1H and PD-L1 in human AML BM core biopsies (right panels) (representative photographs, monocytic AML). Original magnification, ×400. Scale bars: 20 mm. (**B**) Pathologic score of PD-1H and PD-L1 expression in AML BM core biopsies. Scores of 0, 1, 2, and 3 indicate that less than 5%, 5%–20%, 20%–40%, and more than 40% of AML blasts, respectively, showed PD-1H or PD-L1 expression. (**C**) Flow cytometric analysis of healthy donor (HD) CD34^+^ cells (far left), AML blasts (either CD34^+^ or CD33^+^) (second panel), HD CD11b^+^ myeloid cells (third panel), and HD CD3^+^ T cells (far right). (**D**) Change in (Δ) MFI (MFI in PD-1H staining–MFI in isotype staining). Mean value of ΔMFI in HD CD34^+^ progenitors versus mean value of ΔMFI in AML CD34^+^ blasts = 76 ± 26.8 (*n* = 5) versus 11,469 ± 4,873 (*n* = 26), *P* = 0.02. *P* value determined by Student’s *t* test. Error bars represent SEM. (**E**) Flow cytometric analysis of AML subsets (t[8;21], complex karyotype, nonmonocytic, and monocytic). (**F**) Mean value of ΔMFI in t(8;21) versus in monosomic complex karyotype AML (551 ± 145 [*n* = 4] versus 9,469 ± 3,880 [*n* = 8]). *P* value determined by 1-way ANOVA. Error bars represent SEM. *P < 0.05. (**G**) Mean value of ΔMFI in nonmonocytic versus monocytic AML (822 ± 155 [*n* = 19] versus 23,881 ± 9,533 [*n* = 7]). *P* value determined by 1-way ANOVA. Error bars represent SEM. *P <0.05.

To determine the expression of PD-1H surface protein in human AML, we evaluated BM core biopsies sampled from 21 AML patients by IHC (Supplemental Table 1). Interestingly, PD-1H surface protein was expressed on AML blasts in BM from 19 out of 21 AML patients (higher than IHC score 1: >5% of blasts) (Figure 1, A and B, Supplemental Figure 2, and Supplemental Table 1). PD-L1 expression, however, was largely minimal on AML blasts (Figure 1, A and B, and Supplemental Figure 2), although we saw weak expression in normal myeloid subsets. Our data somewhat contradict previous reports that demonstrated PD-L1 expression in myeloid leukemia (62, 63). But of note, these prior data were based on mRNA expression of PD-L1 compared with our assay to detect PD-L1 protein. These data suggest that PD-1H may be one of the important immune modulators in AML. Among subtypes of AML, complex karyotype AML had higher cell-surface expression of PD-1H than t(8;21) and t(15;17) AML, suggesting poor-risk AML, such as complex karyotype, tends toward higher expression of PD-1H than favorable-risk AML, such as t(8;21) and t(15;17) (Supplemental Figure 2). More obviously, PD-1H expression was significantly higher in monocytic AML than nonmonocytic AML (Supplemental Figure 2). These data are consistent with TCGA mRNA expression data (Supplemental Figure 1, B–E).

We confirmed that PD-1H cell-surface staining in IHC analysis is specific by flow cytometry based on positive control (HL-60–PD-1H), negative control (HL-60–mock), and isotype control staining (Supplemental Figure 3A). The specificity of PD-1H staining was also validated using several different clones of anti-human PD-1H (hPD-1H) mAb and different staining protocols (e.g., fixation or nonfixation prior to staining). Among 3 anti-hPD-1H mAbs, 1 clone, MIH65, provided specific staining before or after fixing cells that allowed us to use this mAb with either fresh, cryopreserved, nonfixed, or fixed AML BM cells for flow cytometric analyses (Supplemental Figure 3, A and B). Consistent with prior reports (42, 44, 52), the flow cytometry data showed that PD-1H surface protein is expressed in normal myeloid cells, but rarely in resting T cells in AML BM (Figure 1C). More importantly, PD-1H was highly expressed on CD34^+^ and CD33^+^ AML blasts in BM from AML patients, consistent with the IHC findings (Figure 1, A and C, Supplemental Figures 2 and 4, and Supplemental Tables 1 and 2). In contrast, normal CD34^+^ progenitor cells in BM from healthy donors exhibited minimal expression of PD-1H cell-surface protein (Figure 1C). We quantified the expression levels of PD-1H cell-surface protein on AML blasts from 25 AML patients to compare with that of CD34^+^ progenitor cells from healthy donors (Supplemental Table 2). The mean fluorescence intensity (MFI) of PD-1H in AML blasts (*n* = 25) was significantly higher than the MFI of PD-1H in normal CD34^+^ progenitors from all healthy donors (*n* = 6) (Figure 1D). Consistent with database analyses of PD-1H mRNA transcript, M4 and M5 AML were the subtypes with higher expression of PD-1H surface protein (Figure 1, E and G, and Supplemental Table 2), and t(8;21) AML blasts had very low expression of PD-1H (Figure 1, E and F, and Supplemental Table 2). We also found that PD-1H expression was higher in monocytic leukemia cell lines (THP1, U937, MOLM14) than in leukemia cell lines containing RUNX1-RUNX1T1 (Kasumi1) and PML-RARα (HL-60, NB40) (Supplemental Figure 5).

Collectively, these data suggest that PD-1H surface protein is highly expressed on AML blasts, but not on normal CD34^+^ progenitor cells; that PD-1H surface expression is higher in monocytic leukemia than in nonmonocytic leukemia and in monosomy or complex karyotype AML than in t(8;21) AML; and that high expression of PD-1H in AML BM results mainly from expression of PD-1H by AML blasts in addition to PD-1H expression on normal myeloid cells.

### AML surface PD-1H induces immune evasion.

**Figure 2:**
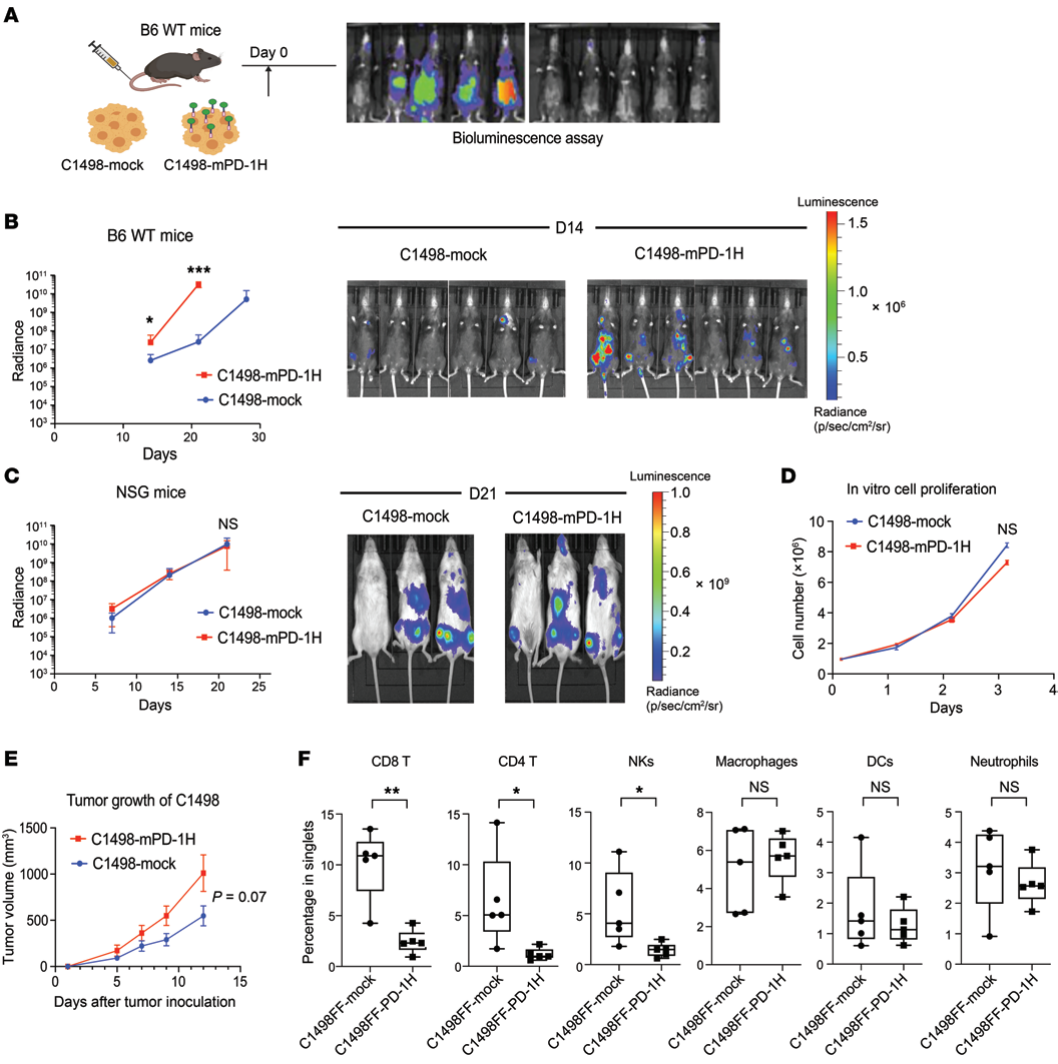
AML surface PD-1H inhibits T cell infiltration, leading to immune evasion. (**A**) Syngeneic mouse leukemia model using tail-vein injection with myeloid leukemia cells (C1498). Mouse leukemia cells expressing PD-1H (C1498FF–PD-1H) or cells not expressing PD-1H (C1498FF-mock) were transplanted into B6 mice and assessed for in vivo leukemia proliferation using bioluminescence. (**B**) In vivo proliferation of C1498FF-mock versus C1498FF–PD-1H cells in B6 WT mice (*n* = 7). Radiance indicates the mean value per group and error bars represent SEM. *P* value determined by Student’s *t* test at each time point. *P <0.05; ***P < 0.001. These experiments were repeated 3 times. Repeated measures were determined by ANOVA with 2 factors (*P* > 0.05, no difference among experiments). (**C**) In vivo proliferation of C1498FF-mock versus C1498FF–PD-1H cells in NSG mice (*n* = 3) (representative images on day 21 on the right side). Radiance indicates the mean value per group, and error bars represent SEM. *P* value determined by Student’s *t* test at each time point. Repeated measures were determined by ANOVA with 2 factors (*P* > 0.05, no difference among experiments). (**D**) In vitro growth of C1498FF–PD-1H tumors compared with C1498FF-mock tumors. Statistical analysis was done using Student’s *t* test. (**E**) Syngeneic mouse model using s.c. injection with C1498 cells. C1498FF–PD-1H cells or C1498FF-mock cells were s.c. injected into the flanks of B6 mice and the tumor volume was assessed. Mean tumor volume ± SEM. *P* value determined by Student’s *t* test at each time point. *n* = 5 per group; *P* = 0.07. Mice were sacrificed on day 12, and tumor tissues were removed for mass cytometry assay. (**F**) Quantification of immune subsets in mass cytometry data in C1498FF–PD-1H tumors compared with C1498FF-mock tumors. *n* = 5 per group, *P* value determined by Student’s *t* test. Error bars represent SEM. *P < 0.05; **P < 0.01.

Since PD-1H expressed on myeloid cells can work as a coinhibitory ligand to negatively modulate T cell activation and function, we hypothesized that PD-1H on the AML cell surface may induce immune evasion. We assessed AML progression in vivo in a syngeneic AML transplant murine model. C1498 is a murine myeloid leukemia cell line that developed spontaneously in a C57BL/6 (B6 hereafter) mouse (64). PD-1H expression in C1498 parental cells is undetectable. We i.v. injected C1498FF cells (engineered to express luciferase) transduced with a PD-1H expression lentiviral plasmid (C1498FF-mouse PD-1H [mPD-1H]) or C1498FF cells transduced with a control lentiviral plasmid (C1498FF-mock) in syngeneic B6 mice (Supplemental Figure 3C) to assess tumor growth in vivo using a bioluminescence assay (Figure 2A). Interestingly, in vivo tumor growth of C1498FF–mPD-1H was significantly faster than that of C1498FF-mock cells in WT B6 mice (mean radiance of C1498FF-mock versus C1498FF–PD-1H on day 21: 2.6 × 10^7^ versus 3.2 × 10^10^, *n* = 7, *P* = 0.0002) (Figure 2B). To determine whether faster in vivo proliferation of C1498FF–mPD-1H cells is associated with immune evasion, we transplanted either C1498FF–mPD-1H or C1498FF-mock cells into immunodeficient NOD-*scid*-IL2Rγ^null^ (NSG) mice. C1498FF–mPD-1H and C1498FF-mock tumors grew equally in NSG mice, suggesting that AML blast PD-1H may promote disease progression by immune evasion (Figure 2C). In addition, these 2 cell lines grew at similar speeds in culture (Figure 2D). Interestingly, we also transplanted C1498FF–mPD-1H or C1498FF-mock cells in PD-1H KO B6 mice and found that, similar to the observation in WT B6 mice, the C1498FF–PD-1H tumor growth was still faster than that of C1498FF-mock cells (mean radiance of C1498FF-mock versus C1498FF–PD-1H on day 21: 1.4 × 10^5^ versus 4.3 × 10^7^, *n* = 7, *P* = 0.01) (Supplemental Figure 6A). These findings suggested that the acceleration of PD-1H^+^ AML in immunocompetent mice is not dependent on PD-1H expression on the host cells.

B7-1 (CD80), a well-known costimulatory ligand, provides a strong antitumor effect via engagement with CD28 on antitumor T cells (65, 66). We transplanted either C1498FF–B7-1 or B7-1/mPD-1H coexpressing (C1498FF–B7-1–mPD-1H) cells into WT B6 mice to assess in vivo tumor growth. Interestingly, C1498FF–B7-1–mPD-1H tumor grew faster in vivo than C1498FF–B7-1 tumor (mean radiance of C1498FF–B7-1 versus C1498FF–B7-1–PD-1H on day 21: 4.6 × 10^5^ versus 5.3 × 10^7^, *n* = 3 per group, *P* = 0.01) (Supplemental Figure 6B). These data suggest that the immune evasion effect of AML blast PD-1H can override the immune activation effect of B7-1.

To facilitate the study of the immune components in a PD-1H–positive versus a PD-1H–negative AML microenvironment, we established a s.c. AML tumor model. Either C1498FF–mPD-1H or C1498FF-mock cells were inoculated s.c. in B6 mice. Consistent with the result when i.v. injected, C1498FF–mPD-1H s.c. tumors also grew faster than C1498FF-mock tumors (Figure 2E) even though the difference was not statistically significant (mean size of C1498FF-mock tumors versus C1498FF–PD-1H tumors on day 12: 547 versus 1,011 mm^3^, *P* = 0.07). The tumors were removed on day 12 after inoculation, and infiltrating immune cells were profiled by mass cytometry (CyTOF), a single-cell analysis tool. C1498FF–mPD-1H tumors had significantly lower immune cell infiltration, especially of CD4^+^ T cells, CD8^+^ T cells, and NK cells. Of note, the infiltration of macrophages and neutrophils in C1498FF–mPD-1H tumors was not significantly different from that in C1498FF-mock tumors (Supplemental Figure 7 and Figure 2F), indicating a selective inhibition by PD-1H on lymphoid cells. To determine whether PD-1H on AML cells suppresses T cells and PD-1H blockade reverses AML PD-1H–mediated T cell inhibition, we transplanted C1498FF–mPD-1H into *PD-1H*-KO mice and treated them with either PD-1H–blocking antibody (13F3) or isotype control. While 13F3 suppressed AML proliferation in vivo, T cell quantity in AML BM and spleen increased in mice treated with 13F3 compared with those treated with isotype control (Supplemental Figure 8).

In addition to overexpressing PD-1H in C1498 cells, we also performed PD-1H knockdown in murine myeloid leukemia cell line WEHI3, which constitutively expresses PD-1H, using shRNA (WEHI3–PD-1H^lo^ versus WEHI–PD-1H^hi^) (Supplemental Figure 3D), and tested the effect of PD-1H knockdown on leukemia growth in vivo. Consistent with the result of the C1498–mPD-1H s.c. tumors, WEHI3–mPD-1H^hi^ tumors grew significantly faster than WEHI3–mPD-1H^lo^ tumors (Supplemental Figure 9A) (*P <* 0.05). Meanwhile, IHC studies suggested that WEHI3–mPD-1H^hi^ tumors have lower infiltration of T cells than WEHI3–mPD-1H^lo^ tumors (Supplemental Figure 9B). Together, these data suggest that AML blast PD-1H induces immune evasion by suppressing infiltrating T cells in the leukemia microenvironment and thereby promotes leukemia growth.

### Host-derived PD-1H also mediates immune evasion in AML.

**Figure 3:**
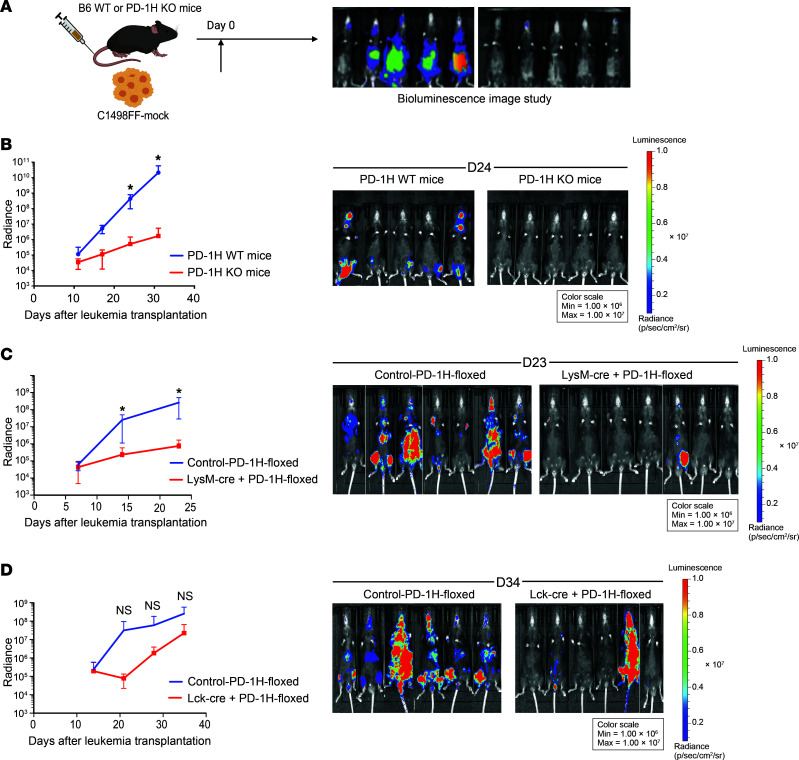
Host-derived PD-1H induces immune evasion in AML. (**A**) Syngeneic mouse leukemia model using tail-vein injection with myeloid leukemia cells (C1498). Mouse leukemia cells (C1498FF-mock) were transplanted into B6 PD-1H WT or *PD-1H*–KO mice or lineage-specific *PD-1H*–KO mice. In vivo proliferation was assessed by bioluminescence. (**B**) In vivo antileukemia effect of genetic deletion of PD-1H in host mice. Radiance indicates the mean value per group, and error bars represent SEM. *P* value determined by Student’s *t* test at each time point. *n* = 5 per group; *P < 0.05. These experiments were repeated 3 times. Repeated measures were determined by ANOVA with 2 factors (*P* > 0.05, no difference among experiments). (**C**) In vivo antileukemia effect of myeloid lineage–specific deletion of PD-1H in host mice. Bioluminescence was compared in LysM-Cre^+^PD-1H-floxed mice with control–PD-1H-floxed mice. Radiance indicates the mean value per group, and error bars represent SEM. *P* value determined by Student’s *t* test at each time point. *P < 0.05. *n* = 9 per group. Representative data from 2 independent experiments were combined. Repeated measures were determined by ANOVA (*P* > 0.05, no difference among experiments). (**D**) In vivo antileukemia effect of T cell lineage–specific deletion of PD-1H in host mice. Bioluminescence was compared in Lck-Cre^+^PD-1H-floxed mice versus control–PD-1H-floxed mice. Radiance indicates the mean value per group, and error bars represent SEM. *P* value determined by Student’s *t* test at each time point. Error bars represent SEM. *n* = 6 per group. Repeated measures were determined by ANOVA (*P* > 0.05, no difference among experiments).

While PD-1H is expressed on AML blasts and acts as a ligand to suppress T cell activation as demonstrated above, PD-1H is also expressed on host immune cells, including T cells and macrophages (42, 44). We hypothesized that PD-1H on host immune cells (immune cell surface PD-1H) may also contribute to immune evasion in AML. To test this, C1498FF-mock cells were i.v. transplanted into *PD-1H*–KO or WT B6 mice, and tumor growth was monitored using bioluminescence in vivo (Figure 3A). The genetic depletion of PD-1H in KO mice conferred significant antileukemic effects (mean radiance in PD-1H WT versus *PD-1H*–KO mice on day 24: 4.4 × 10^8^ versus 5.1 × 10^5^, *n* = 5, *P* = 0.04) (Figure 3B). This led to improved survival compared with PD-1H WT mice (median survival of PD-1H WT mice versus *PD-1H*–KO mice: 33 versus 65 days, *P* = 0.006) (not shown). BM and spleen from *PD-1H*–KO or WT AML mice were assessed for the quantity of immune cell subsets. The quantities of macrophages and granulocytes were significantly increased in *PD-1H*–KO AML mice compared with WT AML mice. In addition, the ratio of proinflammatory macrophages to antiinflammatory macrophages was higher in *PD-1H*–KO mice than in WT mice (Supplemental Figure 10, A and B). The quantities of macrophages and granulocytes or the ratios of proinflammatory macrophages to antiinflammatory macrophages were not significantly different between naive *PD-1H*–KO and WT mice (Supplemental Figure 10C). Other cell subsets, including regulatory T cells (CD4^+^CD25^hi^FoxP3^+^), and CD4^+^ and CD8^+^ T cells, were not changed while NK cells increased and dendritic cells decreased in PD-1H spleen (Supplemental Figure 10, A and B). The antileukemia effect of host immune PD-1H deletion was recapitulated in PD-1H WT mice treated with anti–mPD-1H mAb (clone 13F3) although this was not statistically significant when compared with isotype control–treated mice (mean radiance of anti–mPD-1H Ab (13F3) versus isotype on day 14: 1.4 × 10^6^ versus 4.4 × 10^7^, *n* = 5, *P* = 0.07) (Supplemental Figure 11).

To further dissect the role of host-derived PD-1H, we generated lineage-specific KO mice that do not express PD-1H in T cells (Lck-Cre^+^PD-1H^fl/fl^ versus Lck-Cre^–^PD-1H^fl/fl^) or in myeloid cells (macrophages, granulocytes) (LysM-Cre^+^PD-1H^fl/fl^ versus LysM-Cre^–^PD-1H^fl/fl^) (Supplemental Figure 12). Following i.v. transplantation with C1498FF cells, we assessed tumor growth in these KO mice and littermate controls using bioluminescence in vivo. We found that the tumor growth was significantly inhibited by myeloid cell–specific genetic deletion of PD-1H, compared with littermate controls (mean radiance in LysM-Cre+PD-1H^fl/fl^ versus LysM-Cre^–^PD-1H^fl/fl^ on day 23: 1.7 × 10^9^ versus 1.7 × 10^6^, *n* = 9, *P* = 0.03) (Figure 3C). T cell–specific genetic deletion of PD-1H showed a trend toward potent antileukemia effects, but it was not statistically significant (mean radiance in Lck-Cre+PD-1H^fl/fl^ versus Lck-Cre^–^PD-1H^fl/fl^ on day 34: 2.5 × 10^8^ versus 2.2 × 10^7^, *n* = 6, *P* = 0.1) (Figure 3D). Taken together, our results support critical roles of both AML blast and host myeloid cell–derived PD-1H on immune evasion to promote AML growth.

### Anti–PD-1H mAb reverses immune evasion in AML.

**Figure 4:**
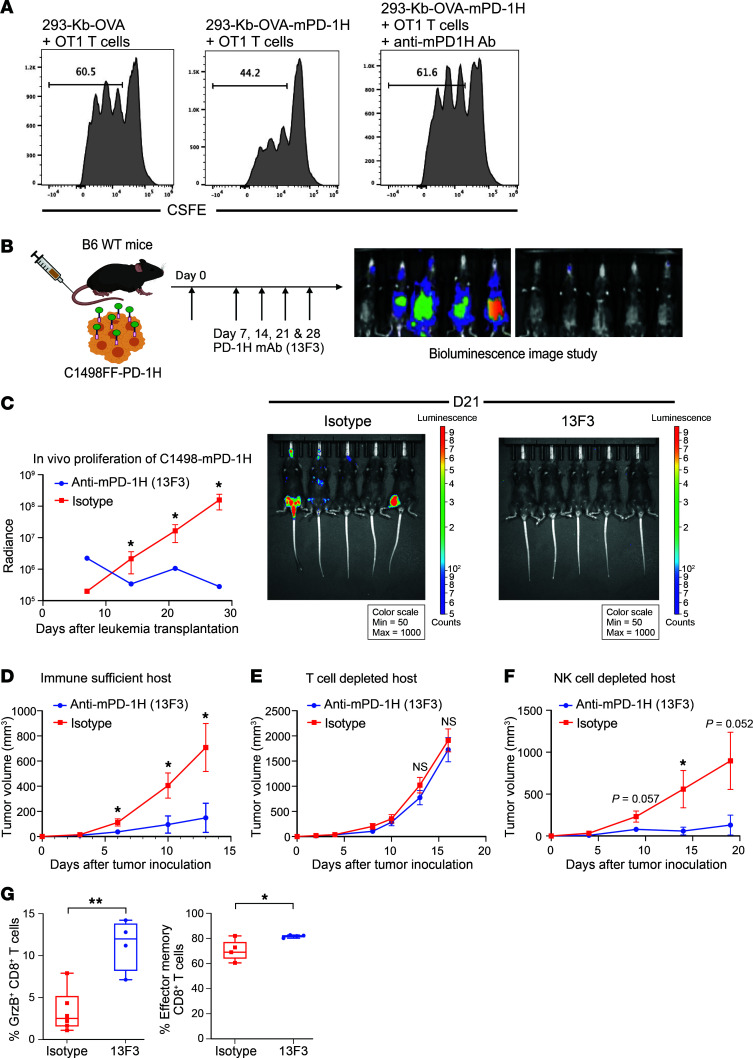
Anti-mouse PD-1H mAb reverses immune evasion induced by mouse AML surface PD-1H. (**A**) PD-1H suppressed T cell activation. Inhibition of OT-1 T cells by mouse PD-1H on 293-K^b^OVA cells. T cell proliferation was assessed by CFSE dilution. The diluted population was assessed by the percentage of total T cells. (**B** and **C**) B6 *PD-1H*–KO mice were transplanted with myeloid leukemia cells expressing full-length PD-1H (C1498FF–PD-1H FL) and treated with anti–mPD-1H mAb (13F3) (**B**). Mice were assessed for in vivo leukemia proliferation using bioluminescence (**C**). A total of 200 μg of 13F3 or isotype control mAb was i.p. injected every 4 days from day 1 of transplantation of C1498FF–PD-1H cells (total 4 doses). Radiance indicates the mean value per group, and error bars represent SEM. *P* value determined by Student’s *t* test at each time point. *n* = 5. *P < 0.05. (**D**–**F**) In vivo growth of C1498FF–PD-1H s.c. tumor in B6 WT mice following anti–mPD-1H mAb treatment. A total of 200 μg of 13F3 or isotype control mAb was i.p. injected every 4 days from day 0 after s.c. injection of C1498FF–PD-1H cells (total 3 doses). (**D**) Tumor size was significantly smaller in the 13F3 treatment group compared with the isotype treatment group. Mean tumor volume ± SEM. Error bars represent SEM. *n* = 6 per group. *P < 0.05. (**E** and **F**) C1498FF–PD-1H s.c. tumor growth with 13F3 or isotype mAb treatment in B6 WT mice depleted of T cells or NK cells. *n* = 6. *P <0.05; ***P <0.01. *P* value determined by Student’s *t* test at each time point. (**G**) Immune cell subsets infiltrated in C1498FF–PD-1H tumors were assessed using mass cytometry. Left: percentages of granzyme B^+^ CD8^+^ T cells in total CD8^+^ T cells. Right: percentages of effector memory phenotype (CD44^+^CD62L^–^) CD8^+^ T cells in total CD8^+^ T cells. *P* value determined by Student’s *t* test. Error bars represent SEM. *P <0.05; **P <0.01.

In the context of an immune-suppressive role of AML blast- and host cell–derived PD-1H, a maximal therapeutic effect may be achieved with a specific mAb to block PD-1H systemically. 13F3 is an mPD-1H–specific mAb that was shown to effectively block the PD-1H pathway and enhance immune responses in mouse tumor and autoimmune disease models (43, 52, 67). We first validated the blocking effect of anti–mPD-1H in an in vitro APC/T cell activation assay. In this assay, a HEK293T-K^b^-OVA cell line (293T-K^b^OVA) stably expressing the mouse H-2K^b^ molecule and the chicken OVA 257–264 peptide (OVA_257–264_) is used as the APC to activate mouse CD8^+^ OT-1 TCR transgenic T cells (68). Compared with 293T-K^b^OVA cells, 293T-K^b^OVA cells stably expressing murine PD-1H on their cell surface (293T–K^b^OVA–PD-1H) induced much less OT-1 T cell proliferation. However, in the presence of 13F3 mAb, 293T-K^b^OVA–PD-1H cells’ inhibitory effect was completely blocked (Figure 4A).

The effect of the PD-1H mAb on AML growth in vivo was first tested using the C1498FF–mPD-1H AML model. After C1498FF–mPD-1H AML cells were transplanted into B6 WT mice either i.v. or s.c., the 13F3 mAb or a control mAb was given to mice. 13F3 treatment dramatically slowed down the in vivo growth of both disseminated C1498FF–mPD-1H AML cells and s.c. tumors (mean radiance of 13F3 versus isotype control on day 28: 2.8 × 10^5^ versus 3.3 × 10^7^, *n* = 5 per group, *P* = 0.02; mean size of C1498FF–PD-1H tumors in mice treated with 13F3 versus with isotype control on day 13: 708.8 versus 148.6 mm^3^, *n* = 6, *P <* 0.05) (Figure 4, B–D). Depletion of T cells by CD4 and CD8 mAbs completely abolished the antileukemic effect of 13F3 in WT B6 mice, whereas NK cell depletion had no effect (Figure 4, E and F). These findings suggest that 13F3 treatment inhibits C1498FF–mPD-1H leukemia growth by enhancing T cell immunity, but not NK cells or antibody-dependent cell–mediated cytotoxicity (ADCC), which is largely mediated by NK cells. Further analysis of T cell subsets in tumor tissues by mass cytometry revealed that the percentages of granzyme B^+^ CD8^+^ T cells as well as effector memory phenotype (CD44^+^CD62L^–^) CD8^+^ T cells were significantly increased, although there was no significant increase of total CD8^+^ or CD4^+^ T cell infiltration in PD-1H–positive leukemia after 13F3 treatment compared with controls (Figure 4G). These data indicate that PD-1H blockade improves the quality of the T cell response rather than augmenting T cell infiltration in this leukemia model.

In the studies described above, we found both AML surface PD-1H and host-derived PD-1H can induce immune evasion in AML. It was unclear whether the therapeutic effect of PD-1H mAb is mediated by either the blocking of PD-1H on AML blasts or PD-1H on the host cells or both. To test the effect of anti–mPD-1H mAbs (13F3) in the absence of host cell–derived PD-1H, C1498FF–PD-1H AML cells were s.c. or i.v transplanted into B6 *PD-1H*–KO mice, and mice were treated with13F3 or control mAbs. We found that 13F3 significantly reduced C1498FF–mPD-1H AML growth in *PD-1H*–KO mice (Supplemental Figure 13, A–C), with an effect similar to that seen in B6 WT mice (Figure 4E). Similar results were also observed using a different anti–mPD-1H mAb (clone mam82) (48) and using another leukemia model (WEHI3) (Supplemental Figure 13, E and F) in *PD-1H*–KO mice, where PD-1H blockade was associated with increased T cell infiltration (Supplemental Figure 13G). To exclude the possibility that the mAb may directly deliver a death signal into AML cells through cell-surface PD-1H, we also assessed in vivo growth of C1498 engineered to express PD-1H without its intracellular domain (C1498FF–PD-1H-Δ). Anti–mPD-1H mAbs could also reduce C1498FF–mPD-1H-Δ growth in vivo, suggesting that mAbs were not affecting signaling within AML cells, but rather blocking the effect of AML blast PD-1H on T cell immune evasion. (Supplemental Figure 13D).

As described earlier, 13F3 treatment had a modest effect on in vivo growth of disseminated C1498FF-mock AML tumors in WT B6 mice (Supplemental Figure 11). These data further confirmed our finding that host-derived PD-1H also contributes to immune evasion in AML. Because C1498 cells do not express PD-1H, the antitumor effect of 13F3 could be attributed to the blockade of host-derived PD-1H.

**Figure 5:**
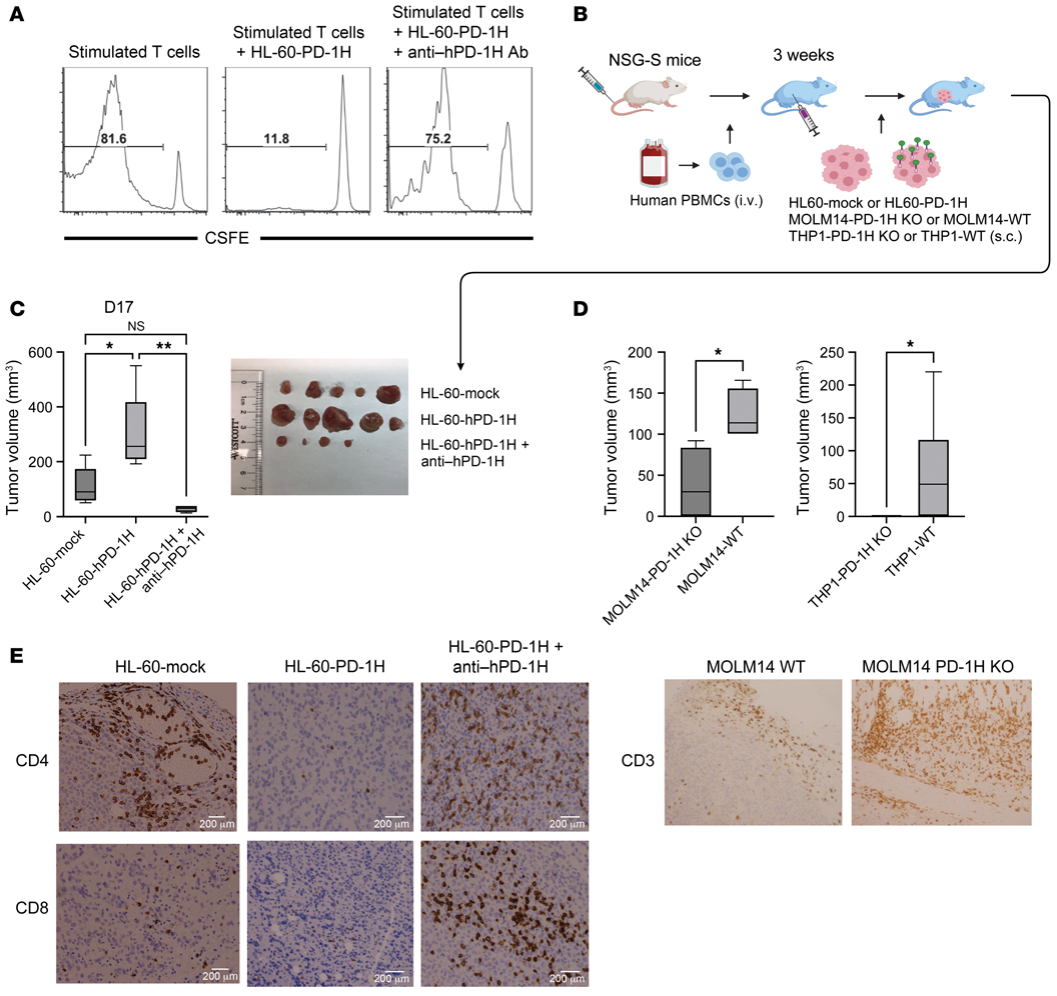
Anti-human PD-1H mAb reverses immune evasion induced by human AML surface PD-1H. (**A**) PD-1H suppressed T cell activation. Inhibition of polyclonal human T cells by human PD-1H on AML (HL-60). T cell proliferation was assessed by CFSE dilution. The diluted population was assessed by the percentage of total T cells. (**B**) The role of human AML PD-1H using a humanized mouse model. Human myeloid leukemia cells expressing PD-1H (HL-60–PD-1H) or not expressing PD-1H (HL-60-mock) were s.c. injected into NSG or NSG-S mice reconstituted with human peripheral blood mononuclear cells. Mice were sacrificed on day 14 and tumor tissues were removed to assess the size and to carry out IHC. (**C** and **D**) The volume of excised leukemia tumors (HL-60, left; MOLM14, middle; THP1, far right) expressing PD-1H or not expressing PD-1H or PD-1H–expressing leukemia tumors following anti-hPD-1H mAb treatment (*n* = 5 per group, *P < 0.05; **P < 0.01). *P* value determined by 1-way ANOVA (**C**) and Student’s *t* test (**D**). Mean tumor volume ± SEM. Error bars represent SEM. Photograph depicts HL-60 tumors removed from humanized NSG-S mice. (**E**) IHC of leukemia tumors expressing PD-1H following anti–hPD-1H mAb to assess CD4^+^ or CD8^+^ T cell infiltration (HL-60) and CD3 (MOLM14).

In addition to the murine AML model, we tested to determine whether human AML blast PD-1H could also induce immune evasion using a humanized AML model. In addition to this mouse T cell activation assay (Figure 4A), we also performed an in vitro human T cell activation/proliferation assay by stimulating human T cells with anti-CD3 mAbs in the presence of HL-60–hPD-1H or HL-60–mock cells. We found that human T cell proliferation was significantly inhibited by PD-1H on HL-60 cells (Figure 5A). Likewise, in the presence of mAbs against hPD-1H (clone MIH65), T cell suppression by HL-60–hPD-1H was reversed (Figure 5A). To determine whether anti–hPD-1H mAbs reverse T cell inhibition induced by PD-1H on human primary AML blasts, we attempted an in vitro T cell activation/proliferation assay in human primary AML BM cells containing PD-1H–expressing blasts. T cell proliferation by polyclonal stimulation with anti-CD3/CD28 was marginal in primary AML BM cells. However, the addition with anti–hPD-1H mAbs induced more significant T cell proliferation (especially CD4^+^ T cells) than isotype control (Supplemental Figure 14).

Using strategies similar to those shown in the murine cell lines, we overexpressed PD-1H in the PD-1H–negative human leukemia cell line HL-60 or knocked out PD-1H in the PD-1H–positive human leukemia cell lines MOLM14 and THP1 (Supplemental Figure 3B). HL-60–hPD-1H or HL-60–mock cells were s.c. injected into immunodeficient NSG-SGM3 (NSG-S) or NSG mice reconstituted with allogeneic human T cells (Figure 5B). Two weeks after leukemia cell inoculation, we sacrificed mice and assessed the size of leukemic tumors. The size of HL-60–hPD-1H tumors (PD-1H^+^) was significantly greater than that of HL-60–mock tumors (PD-1H^–^) (Figure 5C). Consistent with these findings, other PD-1H^+^ AML tumors (MOLM14-WT, THP1-WT) also grew larger than PD-1H^–^ AML tumors (MOLM14–PD-1H KO, THP1–PD-1H KO) (Figure 5D). At the same time, IHC studies showed fewer infiltrating T cells within HL-60–hPD-1H tumors and MOLM14-WT tumors than in HL-60–mock and MOLM14–PD-1H KO tumors, respectively (Figure 5E). But we could not assess T cell infiltration in THP1–PD-1H KO tumors because all tumors were rejected. We determined the effect of an anti-hPD-1H mAbs in a humanized AML model (Figure 5B). The treatment with anti–hPD-1H mAbs (clone MIH65) significantly reduced the size of HL-60–hPD-1H tumors (Figure 5C), which was accompanied by increased T cell infiltration (Figure 5E). Therefore, our findings further extend and validate the results in syngeneic mouse leukemia models showing that PD-1H mAb can reverse the immune evasion induced by PD-1H.

### PD-1H blockade confers a synergistic antileukemic effect with PD-1 blockade.

**Figure 6:**
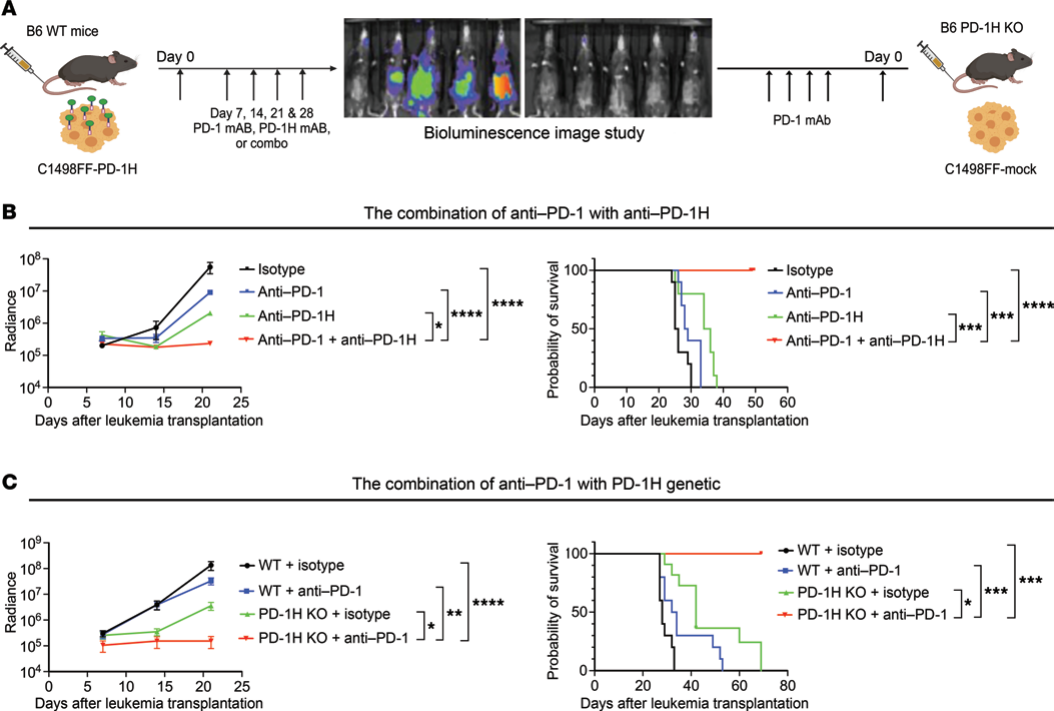
Mouse PD-1H blockade confers a synergistic antileukemic effect with mouse PD-1 blockade. (**A**) Syngeneic mouse leukemia model using tail-vein injection with mouse myeloid leukemia cells expressing PD-1H (C1498FF–PD-1H) transplanted into B6 mice, which were then treated with anti–PD-1 and/or anti–PD-1H mAbs. Syngeneic mouse leukemia model using tail-vein injection with mouse myeloid leukemia cells not expressing PD-1H (C1498FF-mock) transplanted into WT B6 mice or *PD-1H*–KO mice, which were then assessed for in vivo antileukemia effect of genetic deletion of PD-1H in host mice with or without anti–PD-1 mAbs. (**B**) Synergistic antileukemia effect of anti–PD-1 mAb with anti–PD-1H mAb. In vivo proliferation was assessed by bioluminescence (left) and survival by a Kaplan-Meier plot (right). Radiance indicates the mean value per group, and error bars represent SEM. Data from 2 experiments were combined (*n* = 10). (**C**) Synergistic antileukemia effect of genetic deletion of PD-1H in host mice (*PD-1H* KO) with anti–PD-1 mAb. In vivo proliferation was assessed by bioluminescence (left) and survival by a Kaplan-Meier plot (right). Radiance indicates the mean value per group, and error bars represent SEM. Data from 2 experiments were combined (*n* = 10). (**B** and **C**) *P* value determined by simple linear regression method for statistical analysis of radiance and log-rank test for survival. *P < 0.05; **P < 0.01; ***P < 0.001; ****P < 0.0001. These experiments were repeated 2 times. Repeated measures were determined by ANOVA with 2 factors (*P* > 0.05, no difference among experiments).

Consistent with the prior preclinical studies in which PD-1 or PD-L1 blockade had an antileukemia effect (69, 70), we also observed modest reduction of in vivo growth of C1498FF–mPD-1H leukemia in WT mice following anti-mouse PD-1 (anti–mPD-1) mAb treatment compared with an isotype control (Figure 6A). Interestingly, when C1498FF–mPD-1H–bearing mice were treated with anti–mPD-1 mAbs along with anti–mPD-1H mAbs, a synergistic antileukemia effect was observed, compared with either anti–mPD-1 mAb or anti–mPD-1H mAb monotherapy (mean radiance of isotype 5 × 10^7^, anti–PD-1 9 × 10^6^, anti–PD-1H 2 × 10^6^, combination of anti-PD1 with anti–PD-1H 2.3 × 10^5^ on day 21, *n* = 10 per group) (Figure 6B). This synergistic antileukemia effect led to longer survival (mean survival for isotype in WT mice, 25.5 days, for anti–PD-1 in WT mice, 28.5 days, for anti–PD-1H, 35 days, for anti–PD-1+anti–PD-1H, undefined; all *P* values compared with anti–PD-1+anti–PD-1H) (Figure 6B). To confirm these data, we transplanted C1498FF-mock cells into *PD-1H*–KO or WT mice (Figure 6A). In this model, PD-1H was absent in host immune cells as well as on AML cells, which was analogous to treatment with effective PD-1H blockade. Following anti–mPD-1 mAb treatment, in vivo AML growth was assessed using bioluminescence. Consistent with the combination treatment with anti–mPD-1H and anti–mPD-1 mAbs, anti–mPD-1 mAb treatment conferred a synergistic antileukemia effect in *PD-1H*–KO mice compared with anti–mPD-1 mAb treatment in WT mice or isotype treatment in *PD-1H*–KO mice and led to longer survival (mean survival for isotype in WT mice, 32 days; for anti–PD-1 in WT mice, 49 days; for isotype in *PD-1H* KO, 60 days; for anti–PD-1 in *PD-1H* KO mice, undefined; all *P* values compared with anti–PD-1 in *PD-1H* KO mice, *P* < 0.05) (Figure 6C). Our results showed a synergistic effect of blocking both PD-1H and PD-1 pathways in this model.

**Figure 7:**
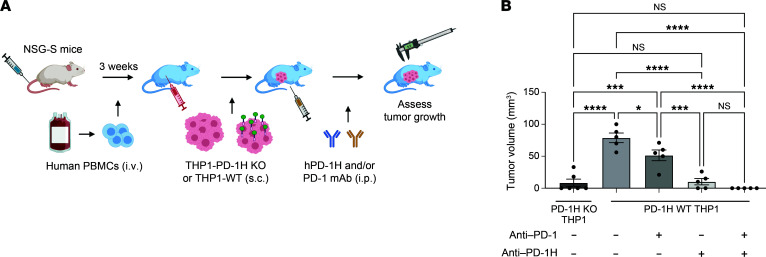
Human PD-1H blockade confers a synergistic antileukemic effect with human PD-1 blockade. (**A**) A humanized AML mouse model to demonstrate a synergistic antileukemia effect of anti–hPD-1 with anti–hPD-1H mAbs. Human myeloid leukemia cells expressing PD-1H (THP1-WT) or not expressing PD-1H (THP1–PD-1H KO) were s.c. injected into NSG mice reconstituted with human peripheral blood mononuclear cells. (**B**) Tumor volume was assessed on days 2, 6, and 9. Anti–hPD-1 (100 μg) and/or anti–hPD-1H mAbs (100 μg) were injected on day 7. Day 9 tumor volume is represented. Mean tumor volume ± SEM. Error bars represent SEM. *n* = 5. *P < 0.05; ***P < 0.001; ****P < 0.001. *P* value determined by 1-way ANOVA.

To test a synergistic antileukemia effect of anti-hPD-1H mAbs with anti-human PD-1 (anti–hPD-1) mAbs, we used a humanized AML model again (Figure 7). THP1-WT (PD-1H^+^) cells were s.c. injected into immunodeficient NSG mice reconstituted with allogeneic human T cells (Figure 7). Following anti–hPD-1H and/or anti–hPD-1 mAbs, we assessed the size of leukemic tumors. Consistent with the observation in Figure 4J, anti–hPD-1H mAbs significantly decreased the size of tumors, but anti–hPD-1 mAbs did not suppress AML tumor growth (Figure 7 and Supplemental Figure 15). Interestingly, the combination of anti–hPD-1H mAbs and anti–hPD-1 mAbs resulted in complete rejection of AML tumors (mean AML tumor volume ± SEM [on day 9] was 44.4 ± 23.3 mm^3^ in THP WT treated with isotype, 33.6 ± 16.8 mm^3^ in THP1 WT treated with anti–PD-1, 14.5 ± 9.8 mm^3^ in THP1 WT treated with anti–PD-1H, 11.1 ± 11.1 mm^3^ in THP1 WT treated with the combination of anti–PD-1 with anti–PD-1H; *n* = 5) (Figure 7). These data suggest that anti–hPD-1H mAb treatment confers a synergistic antileukemia effect with anti-hPD-1 mAbs in human AML.

## Discussion

In this report, we provide evidence that AML blast PD-1H is inhibitory for intrinsic T cell–mediated immune responses against AML and therefore may contribute to escape of AML from immune destruction. We also demonstrate that PD-1H on immune myeloid cells in AML BM may contribute to immune evasions. In this context, blockade of PD-1H by a specific mAb to eliminate its function could improve anti-AML immunity and induce the regression of AML. Finally, we show that, while the effect of PD-1 blockade is modest in a syngeneic AML mouse model and a humanized AML mouse model, combination PD-1/PD-1H blockade confers a synergistic antileukemia effect, leading to the regression of established AML. Our findings provide experimental evidence showing the role of PD-1H in inhibiting anti-AML immunity and implicating a potential new target for AML immunotherapy.

Anti–PD-1 therapy showed unprecedented therapeutic effects on subsets of many different cancers, mainly in solid tumors (34). Early data from clinical trials did show marginal clinical response in myeloid malignancies, such as AML or myelodysplastic syndrome (MDS), when using mAbs targeting CTLA4 or PD-1/PD-L1 as single agents (37, 38, 71, 72). Since hematologic malignancies do not have an obvious tumor immune microenvironment (TIME) as solid tumors do (19, 73, 74), the underlying immune evasion mechanisms for the poor response to immune checkpoint blockades in AML/MDS could be different. Recently, Williams et al. showed that T cells are present and phenotypically changed in the AML BM and that the phenotype bears similarity to the exhausted or persistently activated phenotype (PD-1^+^, OX40^+^, TIM3^+^, LAG3^+^) seen in other cancers (40). A study by Lamble et al. suggests that anti–PD-1 mAb converted “exhausted” T cells back to active effector cells in AML ex vivo (75). Therefore, the marginal clinical response to anti-PD therapy in AML might be associated with in vivo tumor-evasion mechanisms other than the PD-1/PD-L1 pathway. Another possibility is that the exhausted T cells are not responsible for immune evasion in AML. Several studies reveal that dysfunctional T cells in cancer may display phenotypes other than exhaustion and that these phenotypes include but are not limited to anergy, ignorance, and burnout (76). PD-1H has been shown to function as both receptor and ligand. As a ligand, it can deliver potent suppressive signals to T cells by shutting down both proximal and downstream T cell receptor signals. We and others showed that PD-1H, upon binding to T cells, decreased phosphorylation of LAT, SLP76, PLCγ-1, Akt, and Erk1/2 (47, 77). Blando et al. showed that PD-1H was superior to PD-L1 in suppressing T cell cytokine release (IFN-γ, TNF-α) when cocultured with pancreatic tumor–infiltrating lymphocytes (55). These results indicate that the PD-1H signaling axis is a powerful immunomodulatory pathway. PD-1H may execute its inhibitory function via its receptor or receptors on T cells; this remains to be fully elucidated. Our recent analysis of PD-1H molecular structure reveals a unique noncanonical immunoglobulin V–like region that may allow multiple binding partner interactions (78). PD-1H appears to bind PD-1H, VSIG3, and more recently, P-selectin glycoprotein ligand-1 (PSGL-1) (49, 51). Interestingly, the binding of PD-1H to PSGL-1 is dependent on acidic pH (49), which is more common in solid tumors, and its role in our system is unknown. This question will be tested in future studies to assess pH, PSGL-1 expression, colocalization of PSGL-1 with PD-1H in human AML BM, and functional assessment of PSGL-1 in AML.

We demonstrate that AML BM has high expression of PD-1H and that PD-1H expression is higher in monocytic and myelomonocytic AML cells than in nonmonocytic AML cells and healthy donor BM. Also, PD-1H expression is higher in poor-risk complex karyotype AML than in t(8;21) and t(15;17) good-risk AML. The differential expression of PD-1H mRNA observed in TCGA AML correlates with the expression of AML surface PD-1H assessed by flow cytometry. For example, PD-1H expression on monocytic blasts is higher than on nonmonocytic blasts and PD-1H expression on complex karyotype AML blasts is higher than on t(8;21) good-risk AML blasts (no data acquired for t[15;17] AML). This suggests that PD-1H targeting can be more effective in monocytic leukemia. It remains to be elucidated what regulates the expression level of PD-1H in different types of leukemia blasts; possibilities include altered signaling, epigenetic modulation, or cytokine-related modulation. We also found PD-1H expression in AML is correlated with poor survival. Worse survival in PD-1H^hi^ AML may result from immune evasion induced by PD-1H, but other confounding factors that affect survival, including cytogenetics and certain genetic mutations, cannot be completely excluded to explain the worse survival in PD-1H^hi^ AML.

We demonstrate the role of AML blast PD-1H on immune evasion in vitro and in vivo in a syngeneic AML model as well as in a humanized mouse model. Our results suggest that AML blast PD-1H acts as a ligand that suppresses T cell activation. It remains to be elucidated whether AML blast PD-1H also suppresses the activation of innate immune cells, such as macrophages, granulocytes, and NK cells. To our knowledge, this is one of the few studies demonstrating that a coinhibitory ligand on AML blasts induces immune evasion and that its blockade reverses immune evasion in AML. In addition, we also show the role of immune cell PD-1H in immune evasion in AML in mice with the full or conditional genetic deletion of PD-1H transplanted with syngeneic AML cells. Interestingly, macrophage/neutrophil PD-1H contributed more significantly to immune evasion in AML compared with T cell PD-1H. Our data represent one of the few studies demonstrating the significance of checkpoint molecules expressed on immune myeloid cells in cancer immune evasion, beyond CD47–SIRP1α and PD-1–PD-L1 (79–81). But our data cannot completely rule out the possibility that this immune evasion in AML is from PD-1H on myeloid-derived suppressor cells, which was recently shown using in vitro experiments (53). In addition, it remains to be investigated whether macrophage/granulocyte PD-1H acts as a ligand to suppress T cell activation or acts as a receptor. Interestingly, the genetic deletion of PD-1H from macrophages/granulocytes alone without T cells did not achieve an optimal antileukemia effect. This suggests that macrophage/granulocyte PD-1H has a baseline immune tolerance, but breaking tolerance in innate immunity by PD-1H blockade is not enough to generate a robust antileukemia effect without adaptive immunity from T cells.

Our study has a couple of potential limitations. First, we used mouse myeloid leukemia cell line C1498. Syngeneic leukemia models using C1498 cells have been widely used to test the antileukemia effects of chemical compounds and immunotherapies (82–85). However, the genetic makeup of this cell line may not be the same as that of human AML because a very low mutation rate in most primary human AML cells was observed (TCGA Research Network, 2013). Even though the data we presented here are proof of concept, they will ideally be validated using better humanized mouse models, such as immune-deficient mice reconstituted with autologous CD34^+^ progenitors followed by transplantation of primary AML blasts from the same patients. These models, however, are difficult due to competition of reconstituted T cells with the engraftment of primary AML cells as well as reactivity of human T cells to murine xenoantigens.

Here, we demonstrate that PD-1H on AML cells induces immune evasion by suppressing T cells and that host immune cell–derived PD-1H induces immune evasion in AML. PD-1H blockade reverses immune evasion, leading to inhibition of AML progression. Our data strongly suggest that PD-1H is an important immune-suppressive molecule in AML that can be targeted in human AML patients.

## Methods

### Patient samples.

BM core biopsies from patients were formalin fixed and paraffin embedded at the Department of Pathology at Yale University and the Department of Pathology, Microbiology, and Immunology at Vanderbilt University Medical Center. Tissue sectioning and IHC staining were performed by the Histology Core Service at Yale University and by the Translational Pathology Shared Resource at Vanderbilt University Medical Center.

### Animals.

*PD-1H*–KO (GenBank gene NM_028732; GenBank protein JN6-01284) mice were purchased from the Mutant Mouse Regional Resource Center at the University of California–Davis. B6 and BALB/c *PD-1H*–KO mice were generated as previously described (42, 48). *PD-1H* WT B6 mice generated from *PD-1H* heterozygotes were bred and maintained in conditions identical to those of *PD-1H*–KO mice and were used as controls. Some WT B6 mice were purchased from Charles River Laboratories to confirm the data. PD-1H^fl/fl^ mice (a gift of Sam W. Lee at Massachusetts General Hospital, Boston, Massachusetts, USA) (86) were crossed with Lck-cre (B6.Cg-Tg[Lck-cre]548Jxm/J) or LysM-cre (B6.129P2-Lyz2^tm(cre)/lfo^/j) mice purchased from the Jackson Laboratory to generate lineage-specific conditional KO mice (T cells or myeloid cells, respectively). NSG and NSG-S (NOD.Cg-*Prkdc^scid^Il2rgtm1Wjl*Tg[CMV-IL3,CSF2,KITLG]1Eav/MloySzJ) mice were purchased from the Jackson Laboratory.

### Cells.

C1498 is a murine myeloid leukemia cell line that developed spontaneously in a B6 mouse. C1498FF is a stable transfectant of C1498 that expresses firefly luciferase (a gift from Bruce Blazar, University of Minnesota, Minneapolis, Minnesota, USA), used to assess in vivo cell proliferation. C1498FF cells were engineered to stably express mouse PD-1H using transduction with lentivirus (pLenti) expressing full-length mouse PD-1H (C1498FF–PD-1H FL) or PD-1H with deletion of its intracellular domain (C1498FF–PD-1HΔ) or with mock lentivirus (C1498FF-mock). WEHI3 is a murine myeloid leukemia cell line that originated from a BALB/c mouse (purchased from ATCC). WEHI3 cells constitutively express PD-1H. WEHI3 cells were engineered for knockdown or KO of PD-1H expression using shRNA targeting the PD-1H transcript (MISSION Library, Sigma-Aldrich) or CRISPR-Cas9 technologies (gRNA with Cas9 protein), respectively. HL-60 and K562 cells are human myeloid leukemia cell lines not expressing PD-1H. HL-60 or K562 cells were engineered to stably express human PD-1H using transduction with lentivirus expressing full-length human PD-1H (HL-60–PD-1H or K562–PD-1H) or a mock lentivirus (HL-60–mock or K562-mock). MOLM14 and THP1 cells are human monocytic leukemia cell lines expressing PD-1H (gift of Martin Carroll, University of Pennsylvania, Philadelphia, Pennsylvania, USA).

### Flow cytometry for staining human PD-1H.

All human cell preparations were more than 95% viable by trypan blue exclusion. Two million thawed or fresh BM mononuclear cells were stained using mAbs conjugated with Pacific blue, FITC, PE-Cy7, PE, PerCP-Cy5.5, APC specific for human CD3, CD11b, CD34, CD33, CD45 (BioLegend), and human PD-1H (VISTA) (clone MIH65, BD Biosciences), respectively, to perform flow cytometry (Supplemental Table 4). After staining, cells were washed, resuspended in PBS with 1% paraformaldehyde, and analyzed in an Attune Flow Cytometer (Thermo Fisher) using FlowJo software (Tree Star).

### Assessment of TMEs using mass cytometry.

B6 mice were inoculated with 3 × 10^6^ C1498FF-mock or C1498FF–PD-1H cells. Mice were sacrificed on day 12, and tumor tissues were removed. Tumor tissue in comparable size (roughly 0.2 gram) from each mouse was used as 1 sample with the following treatment. Tumor tissue was homogenized and digested with collagenase IV (200 μg/mL) and DNase (20 μg/mL) for 30 minutes before tissue dissociation using the gentleMACS Dissociator (Miltenyi Biotec). Single-cell suspensions with 5 × 10^6^ total cells were then incubated with the mAb against mouse CD16/CD32 for 10 minutes at room temperature to block Fc receptors and subsequently stained with the metal-labeled mAb cocktail against cell-surface molecules per the protocol described in Supplemental Methods (Supplemental Table 3).

### Myeloid leukemia model for in vivo imaging analyses.

Approximately 3 × 10^5^ of C1498FF–PD-1H-FL, C1498FF–PD-1HΔ, or C1498FF-mock cells in 300 μL PBS were i.v. injected into B6 WT or *PD-1H*–KO mice. To assess in vivo proliferation of C1498FF cells, mice were i.p. injected with 300 μg luciferin substrate 5 minutes prior to being anesthetized using an XRT-8 gas (isoflurane) anesthesia system. Anesthesia was maintained while mice were imaged for bioluminescence in a supine position using an IVIS Lumina XR In Vivo Imaging System (Caliper/PerkinElmer) according to the manufacturer’s protocol. Briefly, luminescence detection was set to automatic with a minimum detection level of 3,000 photons. Mice were imaged on stage D at 1.5 cm height from the stage. Units were set to radiance (photons/s). Imaging and analysis were performed using Living Image software (version 4.7.4). For analysis, binning was set to 4, and minimum and maximum radiance levels were determined for optimal view and comparison between groups at each time point. Calculation of total flux was assessed (radiance or photons/s) in each pixel and then summed or integrated over the whole body (cm^2^ × 4π) by Living Image software. For experiments to evaluate the efficacy of PD-1H blockades, 3 × 10^5^ of C1498FF-mock or C1498FF–PD-1H-FL cells in 300 μL PBS were i.v. injected into B6 WT or *PD-1H*–KO mice. We assessed in vivo proliferation of AML cells following i.p. injection of 200 μg of anti–PD-1H mAb (clone 13F3) or hamster IgG (BioXcell) on days 0, 7, 14, and 21 after AML cell i.v. injection. For experiments to evaluate the combination efficacy of PD-1 and PD-1H blockades, C1498FF–PD-1H-FL cells in 300 μL PBS were i.v. injected into B6 WT mice. We assessed in vivo proliferation of AML cells following i.p. injection of 200 μg of anti–mPD-1H mAb (clone 13F3) and/or anti–mPD-1 mAb (clone RMP1-14) or hamster IgG (BioXcell) on days 0, 7, 14, and 21 after AML cell i.v. injection. For other experiments for combination efficacy of PD-1 and PD-1H blockades, C1498FF-mock cells in 300 μL PBS were i.v. injected into B6 WT or *PD-1H*–KO mice. We assessed in vivo proliferation of AML cells following i.p. injection of 200 μg of anti–mPD-1 mAb (clone RMP1-14) or hamster IgG (BioXcell) on days 0, 7, 14, and 21 after AML cell i.v. injection. We repeated these experiments at least 2 or 3 times and found data were reproducible.

### Myeloid leukemia s.c. model.

B6 WT or *PD-1H*–KO mice were inoculated s.c. in the right flank with 3 × 10^6^ C1498FF-mock or C1498FF–PD-1H cells. BALB/c mice were inoculated s.c. in the right flank with 0.5 × 10^6^ WEHI3–PD-1H shRNA or WEHI3-control shRNA cells. Tumor size was monitored every 5 days. Tumor volume was calculated as volume (mm^3^) = width (mm) × length (mm) × ½ width (mm). To test the effect of anti–PD-1H mAb on tumor growth, 200 μg of anti–mPD-1H (clone 13F3) mAb or hamster IgG (BioXcell) was i.p. injected on days 0, 4, and 8 after C1498FF–PD-1H tumor inoculation.

### In vivo immune cell depletion.

To deplete T cells, 250 μg of anti-CD4 (clone GK1.5) and 250 μg of CD8α (clone 53-6.7) were injected on days –4, –2, 2, 6, and 10 around tumor inoculation. To deplete NK cells, 500 μg of anti-NK1.1 (clone PK136) was injected 2 days before tumor inoculation, followed by 3 doses of 250 μg on postinoculation days 2, 6, and 10.

### Humanized myeloid leukemia mouse model.

Approximately 5 × 10^6^ human peripheral blood mononuclear cells were transplanted into NSG-S or NSG mice. After 3 weeks of transplantation, the engraftment of human cells was confirmed by human CD45 using flow cytometry. Either 1 × 10^6^ HL-60–hPD-1H or HL-60–mock cells (or 4 × 10^6^ THP1-WT or THP1–PD-1H KO; MOLM14 WT or MOLM14–PD-1H KO) were s.c. injected into the flanks of the immune reconstituted NSG-S or NSG mice. Anti-human PD-1H mAbs (clone MIH65) or anti-human PD-1 mAbs (pembrolizumab) or isotype control Abs were i.p. injected weekly from the day of tumor injection. Tumor volume was calculated as volume (mm^3^) = width (mm) × length (mm) × ½ width (mm). Tumors were removed from euthanized mice to evaluate immune-cell infiltration. We repeated these experiments at least 2 or 3 times and found data were reproducible.

### In vitro mouse OT-I CD8^+^ T cell activation by HEK293T-Kb-OVA cell lines.

OT-I T cells were purified from lymph nodes and spleen of Rag1KO/OT-I mice (Taconic) with the EasySep Mouse CD8^+^ T Cell Isolation Kit (STEMCELL Technologies) and labeled with 5 μM CFSE. Next, 2 × 10^5^ OT-I cells were cocultured with 4 × 10^4^ UV-radiated 293TKbOVA or 293TKbOVA–mPD-1H cells in a 96-well flat bottom plate (Corning). Anti-mouse PD-1H blocking antibody 13F3 or control hamster IgG (Bio -X Cell) was added into culture at 5 μg/ml as a final concentration. Three days later, cells were harvested and stained by anti-CD8 (BD). CFSE profiles on the CD8^+^ gate were analyzed on Attune NxT Cytometer (Life Technology).

### Leukemia cell and T cell coculture assay.

Human T cells were purified from peripheral blood mononuclear cells or whole blood using an EasySep Human T Cell Isolation Kit (STEMCELL Technologies). Purified T cells were labeled with CFSE (Thermo Fisher). These T cells (4 × 10^4^) were mixed with irradiated HL-60–hPD-1H or HL-60–mock cells with anti-human PD-1H mAbs (clone MIH65) or isotype control (500 μg/mL) in a U-bottom 96-well plate. The E/T ratio was typically 4:1. Immunocult Human CD3/CD28 T Cell Activator (25 μL/mL) (STEMCELL Technologies) and recombinant human IL-2 (50 U/mL) were added to stimulate T cells. Cells were assessed for CFSE dilution using flow cytometry.

### Statistics.

Graphs and statistical analyses were generated with GraphPad Prism 6 (GraphPad Software). Statistical analyses of survival experiments were performed using a log rank (Mantel-Cox) test; all other analyses were performed using an unpaired *t* test with Welch’s correction and a linear regression. The nonparametric Kruskal-Wallis test followed by Dunn’s multiple-comparisons test was used for identifying differences between groups in the TCGA data set. *P* <0.05 was considered significant.

### Study approval.

All patients gave informed consent to participate in this study, which had the approval and guidance of the Institutional Review Boards at Yale University (no. 12642) and Vanderbilt University Medical Center (IRB no. 192382). BM aspirate samples were coded and processed by the Hematology Tissue Bank at Yale University and the Hematology Tissue Bank at Vanderbilt University. All mouse procedures were performed in accordance with institutional guidelines at Yale University (no. 11387) and Vanderbilt University (no. M1900082-01). Mice were maintained according to NIH animal care guidelines, and experimental protocols described in this study were approved by Yale University’s and Vanderbilt University’s Institutional Animal Care and Use Committees.

### Data availability.

Values for all data points in graphs are reported in the Supporting Data Values file. Data are available upon request.

## Author contributions

TKK conceived, conceptually designed, and supervised the study; carried out experiments; performed data analysis; and was a primary author of the manuscript. XH conceptually designed the study, carried out experiments, performed data analysis, and was a primary author of the manuscript. QH designed the study, carried out experiments, performed data analysis, and contributed to writing the manuscript. ENV carried out experiments, performed data analysis, and contributed to writing the manuscript. CMF, JH, and KWK carried out experiments and performed data analyses. EFM and RSP assessed IHCs and contributed to writing the manuscript. JW, QW, JPZ, TB, MFS, LZ, TZ, JA, SWL, and NSC carried out experiments and performed data analyses. AMZ, SH, MMP, JL, MLX, and SDG provided scientific insight and contributed to writing the manuscript. LC conceptually designed and supervised the study, performed data analysis, and contributed to writing the manuscript.

## Supplementary Material

Supplemental data

Supporting data values

## Version 1 12/07/2023

In-Press Preview

## Version 2 02/01/2024

Electronic publication
