# Supplementary material for: PD-1H/VISTA mediates immune evasion in acute myeloid leukemia
Source: J Clin Invest. 2024 Feb 1;134(3):e164325. doi: 10.1172/JCI164325 (PMC10836799; doi:10.1172/JCI164325)
Supplement: Supplemental data [file jci-134-164325-s236.pdf]

Supplemental Methods

Database analyses

Normalized RNA-seq by Expectation and Maximization (RSEM) mRNA expression data on 162 samples from AML patients included in the Cancer Genome Atlas (TCGA) project were downloaded from www.cbioportal.org (hosted by Memorial Sloan Kettering Cancer Center) together with information on corresponding clinical, mutational and cytogenetic parameters. The PD-1H (C10orf54) expression pattern in human cancer tissues was evaluated using TCGA cancer databases.

Immunohistochemistry

To perform IHC, tissue sections (5 μm thick) from formalin-fixed, paraffin-embedded blocks of BM core biopsies were prepared per protocol. Tissue sections were then incubated with rabbit anti-hPD-1H mAb (clone D1L2G, Cell Signaling Technology) and rabbit anti-hPD-L1 (PA5-28115, Thermo Fisher) for 30 min and subsequently with anti-rabbit IgG conjugated with horseradish peroxidase at room temperature. All tissue sections were counterstained with hematoxylin and eosin (H&E) for 2 min. Slides were then air dried and cover-slipped. PD-1H and PD-L1 expression was evaluated by pathologists at Yale University and Vanderbilt University Medical Center. Pathologic scores of 0, 1, 2, and 3 indicate that <5%, 5-20%, 20-40%, and >40% of of AML blasts, respectively, showed PD-1H or PD-L1 expression.

Flow cytometry analysis for human PD-1H expression in AML

For most of the analyses, at least $3x10^5$ total events were analyzed, with sequential gating of BM mononuclear cells in different cell regions (by CD45 and side scatter). Each region was assessed as CD34 and/or CD33, CD3 and CD11b to demarcate blasts, and T cells and

non-blast myeloid cells staining for PD-1H compared with isotype control. We used HL60-PD-1H and HL60-mock cells to validate the specificity of PD-1H mAbs (clone MIH65 (BD Biosciences); clone 730804 (R&D Systems, Minneapolis, MN); clone D1L2G (Cell Signaling Technology, Danvers, MA); clone 1H11 (generated by ourselves)). We used several human AML cell lines including Kasumi1, NB4, HL-60, U937, HEL1 (gifts from Manoj Pillai, Yale University), and THP1 and MOLM14 (gifts from Martin Carroll, University of Pennsylvania) to assess PD-1H expression using MIH65 by flow cytometry. We also assessed PD-1H expression in murine AML cells using rat anti-mouse PD-1H (clone MIH64 (BD Biosciences)) by flow cytometry.

Assessment of tumor microenvironments using mass cytometry

After tumor dissociation, cells were resuspended with RPMI 1640 and 10 µM cisplatin (Fluidigm) in a total volume of 400 µL for 60 s before quenching 1:1 with pure fetal bovine serum to determine viability. Cells were centrifuged at 600×g for 7 min at 4°C and washed once with PBS with 0.5% bovine serum albumin (BSA) and 0.02% NaN_3. Cells were then fixed using Fixation/Permeabilization Buffer (ebioscience) for 30 min at 4°C. After the treatment with the Fixation/Permeabilization Buffer, cells were further incubated with the mAb cocktails against intracellular proteins. Metal-conjugated antibodies were purchased from Fluidigm or from BioLegend and conjugated in the laboratory. Then, cells were washed twice in PBS with 0.5% BSA and 0.02% NaN_3, followed by staining with 1 mL of 1:4000 191/193Ir DNA intercalator (Fluidigm) diluted in PBS with 1.6% paraformaldehyde overnight. The day after, cell samples were diluted in ddH_2O containing bead standards to about 10^6 cells/mL and then acquired on a mass cytometer (Helios, CyTOF 3, Fluidigm) equilibrated with ddH_2O. Data were analyzed using Cytobank.

Assessment of tumor microenvironments using flow cytometry

Mouse spleen, bone marrow (BM) and blood were harvest for flow cytometric analysis. Mouse spleens were minced and pressed through a 70-µm strainer using a syringe plunger to obtain a single-cell suspension. Bone marrow was flushed with 5% FBS RPMI, centrifuged, resuspended, and passed through a 70-µm strainer for single-cell suspension. Erythrocytes were lysed by incubation in red cell lysis buffer (eBioscience) for 5 min (spleen and BM) at room temperature. Briefly, cells were incubated with LIVE/DEAD™ Fixable Aqua Dead Cell Stain Kit (Invitrogen) to discriminate live/dead cells and washed with FACS buffer (PBS containing 1% v/v FBS). After blocking Fc receptors with anti-mouse CD16/CD32 mAb (BD Biosciences) in FACS buffer for 20 min, cells were incubated with target antibodies (table 2). After staining, cells were washed twice in FACS buffer and fixed with 300 µl/tube of fixation buffer (4% PFA in PBS). Data were collected using a BD LSR Fortessa flow cytometer and analyzed using FlowJo software.

Cells

WEHI3 is a murine myeloid leukemia cell line that originated from a BALB/c mouse (purchased from ATCC). WEHI3 cells constitutively express PD-1H. WEHI3 cells were engineered for knockdown or KO of PD-1H expression using shRNA targeting the PD-1H transcript (the Mission Library, Sigma) or CRISPR-Cas9 technologies (gRNA with Cas9 protein), respectively.

For KO via CRISPR, gRNA was designed based on an algorithm of idtdna.com and *ex vivo* combined with Cas9 protein (IDTDNA) according to the manufacturer's recommended protocol before transfecting cells with this ribonucleoprotein complex. For MOLM14 and THP1 cells, we used Amaxa nucleofection, and for WEHI3 cells, we used Neon electroporation.

Generation of HEK293T-Kb-OVA Cell Lines

Full- length mouse PD-1H, including its native signal peptide, was inserted into the pLenti7.3/V5-TOPO-GFP lentivector upstream of the C-terminal V5 tag (Thermo Fisher).

Lentivirus was generated with mouse PD-1H lentivector and ViraPower™ HiPerform™ Lentiviral Expression kit (Thermo Fisher) in HEK293T cells. HEK293T-KbOVA (293T-KbOVA) cell line was transduced with lentivirus carrying mPD-1H gene. Cells were stained by anti-mouse PD-1H monoclonal antibody (mam82 clone, made in our laboratory), and mPD-1H^{+} cells were sorted by BD FACSAriaII. Polyclonal stable cell line 293T-KbOVA-mPD-1H were maintained after sorting.

Primary AML cells and T cell co-culture assay

Primary human AML BM cells were thawed and labeled with CFSE. Anti-human CD3 mAb (OKT3, 2μg/ml) was coated to stimulate T cells in the presence or absence of soluble anti-human PD-1H (MIH65) and/or anti-human PD-1 (Pembrolizumab) or isotype control (5ug/ml). T cells were assessed for CFSE dilution using flow cytometry

Western blotting

Cells were added to lysis buffer (200 μL, BioRad Laemmli buffer including β-mercaptoethanol), and boiled for 10 min at 95-100°C. Equal amounts of protein were loaded into each well and separated on a NuPAGE 4–12% Bis-Tris gel, followed by transfer onto PVDF membranes (Invitrogen). Membranes were blocked with 5% non-fat milk in TBS-T for 1 h at room temperature. The blots were then incubated with antibodies against PD-1H/VISTA (Cell Signaling Technology) overnight at 4°C. Anti-rabbit IgG-HRP (1:1000; KwikQuant) was then added and the mixture incubated for 45 min at room temperature. Immunoblots were developed using the Ultra HRP Substrate solution (KwikQuant). Chemiluminescence imaging was performed using a KwikQuant imager. GAPDH was used as the loading control.

Supplemental figure 1

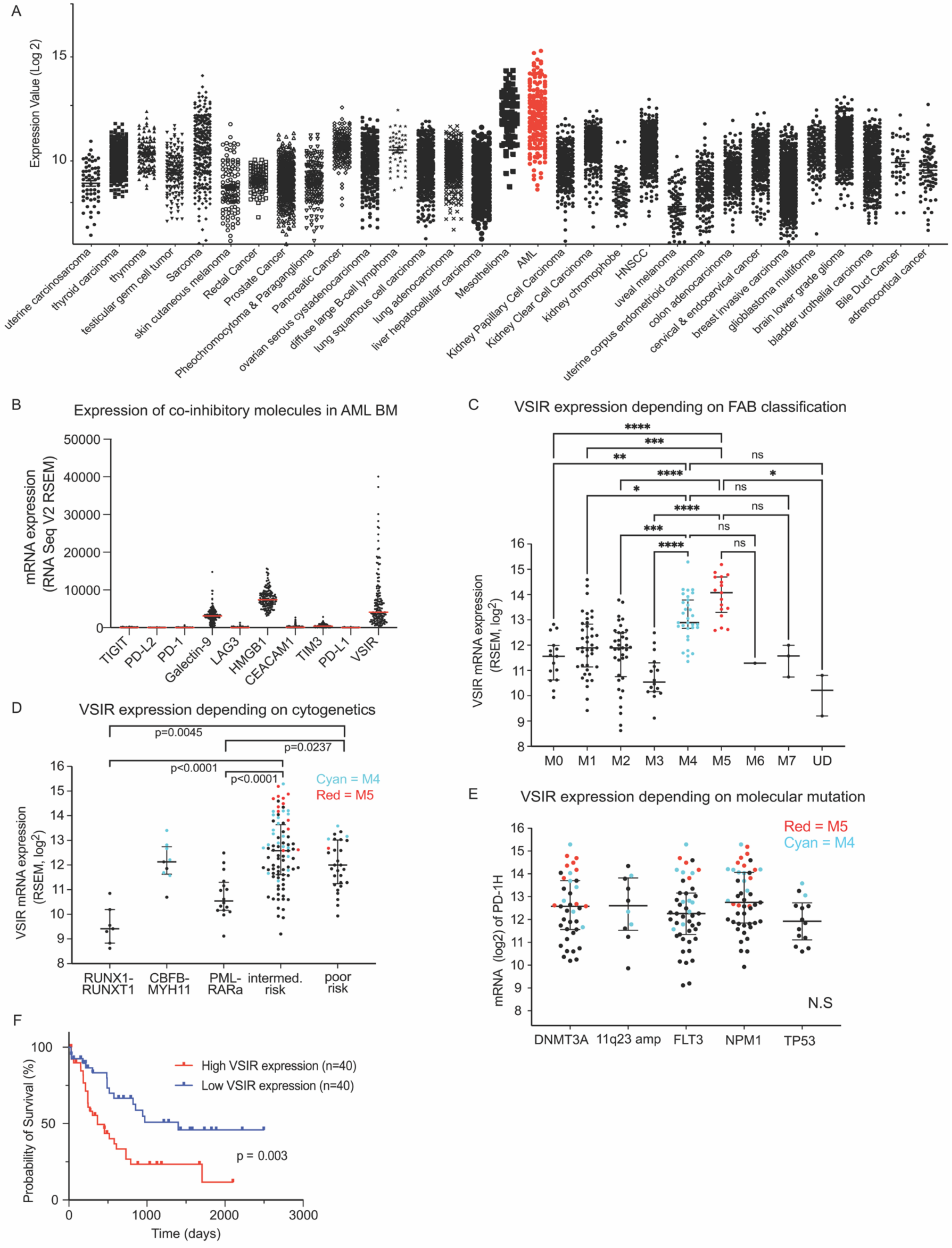

Supplemental Figure 1. *VSIR* mRNA is highly expressed in AML.
A. *VSIR* mRNA expression levels in the indicated human cancers from the TCGA cancer database. AML had the highest level of *VSIR* mRNA among various cancer types.
Normalized RNA-seq by Expectation and Maximization (RSEM) mRNA expression data on 162 samples from AML patients included in the TCGA project together with information on corresponding clinical, mutational, cytogenetic parameters, and survival (B, C, D, E). Cyan and red indicate monocytic leukemia (M4 and M5, respectively) in B, C, D.
B. The expression of co-inhibitory molecules in AML BM.
C. *VSIR* expression in AML French-American-British classification. Monocytic leukemia (M4, M5) had higher expression of *VSIR* than other subtypes of AML (mean of *VSIR* mRNA expression (RSEM, log2) ± SEM: 10,433±1,335 (M4), 17,989±2,297(M5) vs. 5,626±821 (M1), 4,298±527 (M2), 2,074±351 (M3), $p<0.01$).
C. *VSIR* expression in AML with different cytogenetics. Poor risk and intermediate risk AML showed higher expression of *VSIR* than good risk AML (RUNX1-RUNX1T1, CBFB-MYH11, PML-RARα) (mean of *VSIR* mRNA expression (RSEM, log2) ± SEM: 847±191 (RUNX1-RUNX1T1), 2,074±351(PML-RARα) vs. 8,878±910 (intermediate risk AML), 5,061±583 (poor risk AML), $p<0.05$; mean of *VSIR* mRNA expression (RSEM, log2) ± SEM: 5,048±918 (CBFB-MYH11) vs. 8,878±910 (intermediate risk AML), 5,061±583 (poor risk AML), $p>0.05$).
D. *VSIR* expression in AML with different mutational status. Mutations commonly occurring in AML (DNMT3A, 11q23 amplification, FLT3 internal tandem duplication, NPM1, TP53) were not significantly associated with *VSIR* expression level ($p>0.05$, N.S.: not significant).
E. Survival analysis of AML patients with high versus low *VSIR* expression using the 25th percentile cut-offs from the TCGA cancer database. AML patients with high expression of *VSIR* (highest quartile) showed worse survival than those with low expression of *VSIR* (lowest quartile) (median overall survival of *VSIR*^{low} AML= 1,401 days vs. 456 days for *VSIR*^{high} AML, $p=0.007$).

Supplemental figure 2

A

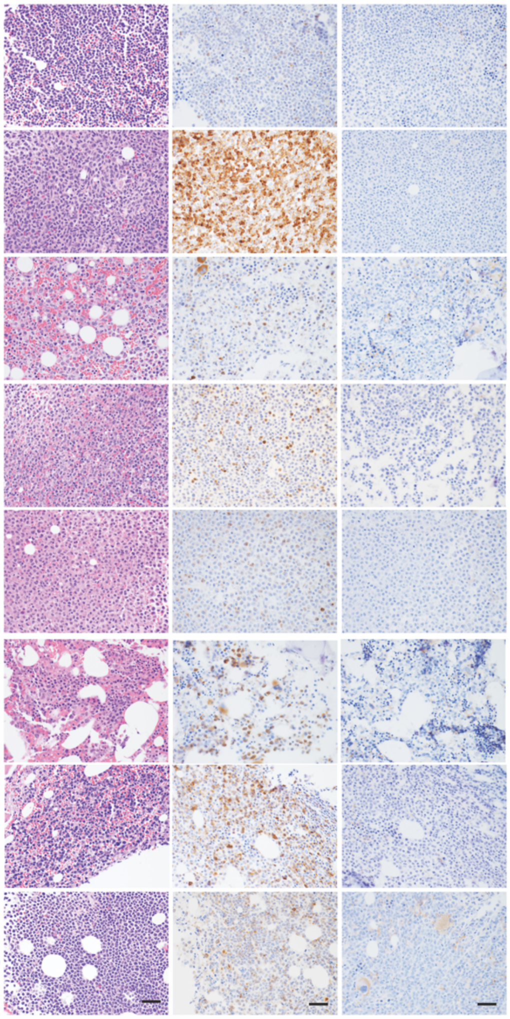

	Blast PD-1H score	Immune cells PD-1H	Blast PD-L1 score	Immune cells PD-L1
AML with biallelic CEBPA mutation	1+	Yes, MK*, PC** (rare cells)	0	Yes, PC (rare cells)
AML with mutated NPM1 (monocytic differentation)	3+	Yes, MK	0	Yes, MK, rare macrophages
AML with t (8;21)	1+	Yes, MK, myeloids	0	Yes, MK, macrophages
AML with inv (16)	1+	Yes, MK, myeloids	0	Yes, MK, macrophages
Acute promyelocytic leukemia	2+	Yes, MK	0	Yes, MK
AML with complex karyotype	3+	Yes, MK	0	Yes, MK
AML with monosomal karyotype	3+	Yes	0	Yes, rare
AML with mutated NPM1 (non-monocytic)	2+	Yes, MK, Mautring myeloids	0	Yes, MK

*MK: megakaryocytes
**PC: plasma cells

B

AML

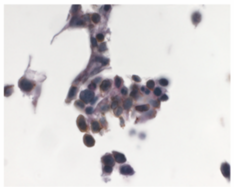

Brown: CD34
Red: PD-1H

Supplemental Figure 2. Immunohistochemical staining of PD-1H and PD-L1 in primary human AML.
A. BM Core biopsies of 7 representative types of AML were stained by H&E (left) or IHC (PD-1H, middle; PD-L1, right). Blast PD-1H and PD-L1 scoring is described in Figure 1. Immune cell PD-1H and PD-L1 expression was also assessed. 400x magnification. Scale bars = 20mm.

B. IHC co-staining of PD-1H and CD34 demonstrating the expression of PD-1H on CD34+ blasts (red: PD-1H, brown: CD34).

Supplemental figure 3

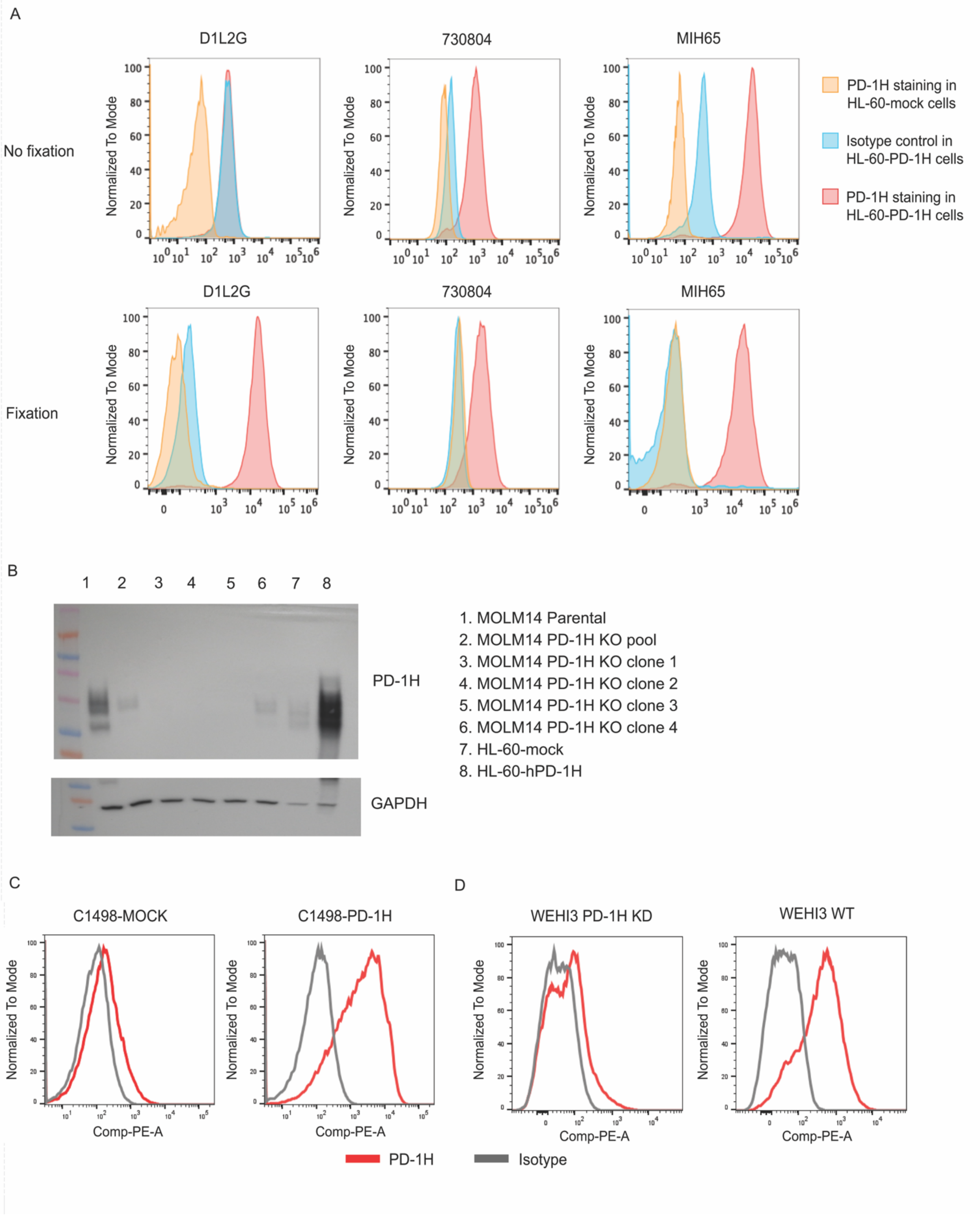

Supplemental Figure 3. Optimizing PD-1H staining in myeloid leukemia cells
A. Optimizing flow cytometry to stain PD-1H. Three antibody clones (D1L2G, 730804, MIH65) were tested in HL-60-PD-1H or HL-60-mock cells. Staining quality was compared between staining before fixation and staining after fixation.
B. Western blotting to demonstrate the specificity of human PD-1H mAb in AML cell lines. PD-1H expression was removed using CRISPR-Cas9 techniques in MOLM14 cells. PD-1H expression in HL-60 parental cells is minimal. HL-60 cells are engineered to express PD-1H (HL-60-hPD-1H) using lentiviral transduction.
C. Engineering to overexpress PD-1H in C1498 cells (C1498FF-mock vs. C1498FF-PD-1H). PD-1H expression was assessed by flow cytometry. Red: PD-1H staining, gray: isotype control.
D. Engineering to knock down PD-1H expression in WEHI3 cells using shRNA (WEHI3 PD-1H KD vs. WEHI3 WT). PD-1H expression was assessed by flow cytometry. Red: PD-1H staining, gray: isotype control.

Supplemental figure 4

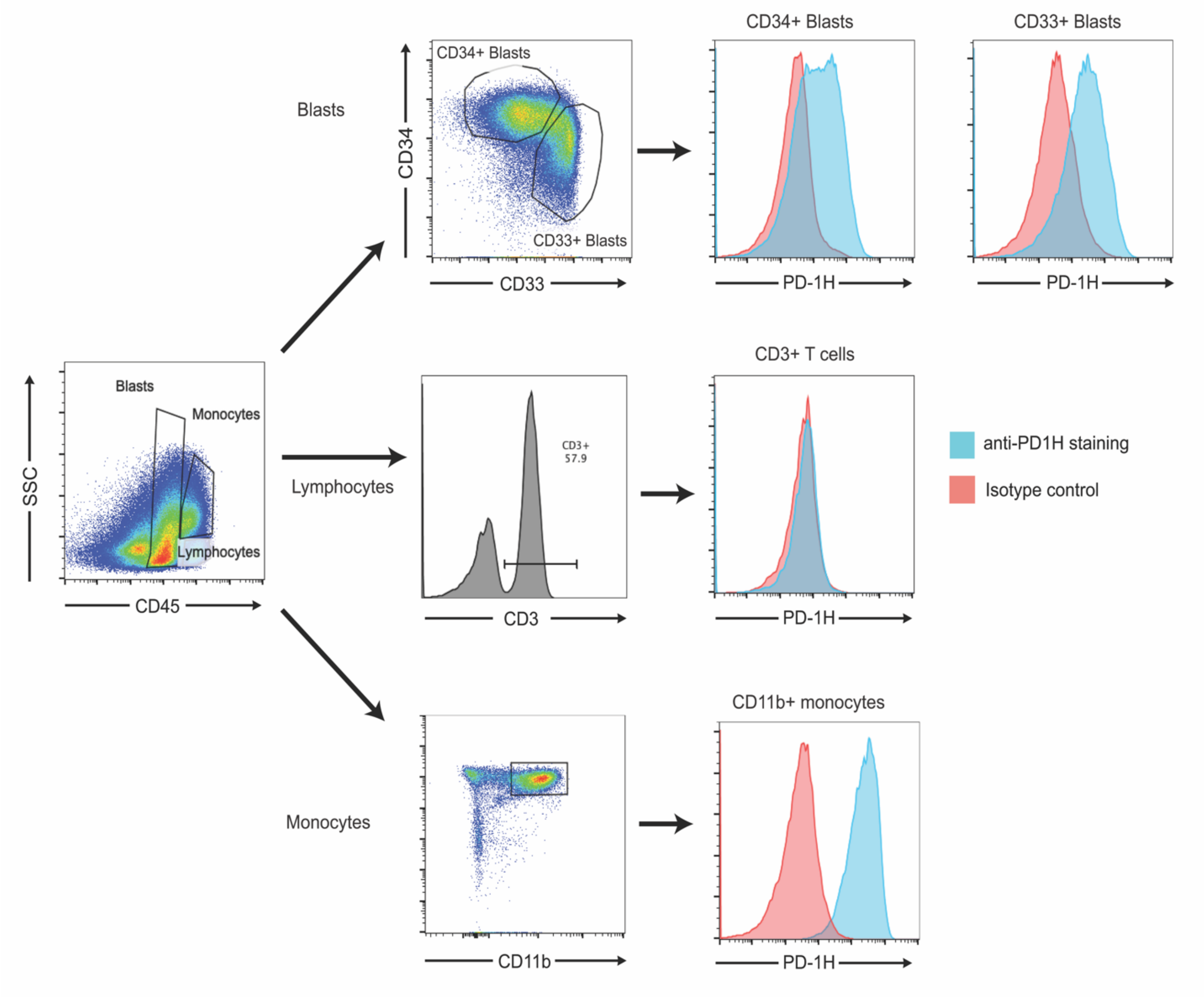

Supplemental Figure 4. Gaiting strategy to assess the expression of PD-1H in human AML BM aspirate.

For most analyses, at least $3x10^5$ total events were analyzed, with sequential gating of BM mononuclear cells in different cell regions (by CD45 and side scatter). Each region (blasts, lymphocytes, monocytes) was further assessed for CD34 and/or CD33, CD3, and CD11b to demarcate blasts, T cells, and non-blast myeloid cells, respectively, staining for PD-1H compared with isotype controls.

Supplemental figure 5

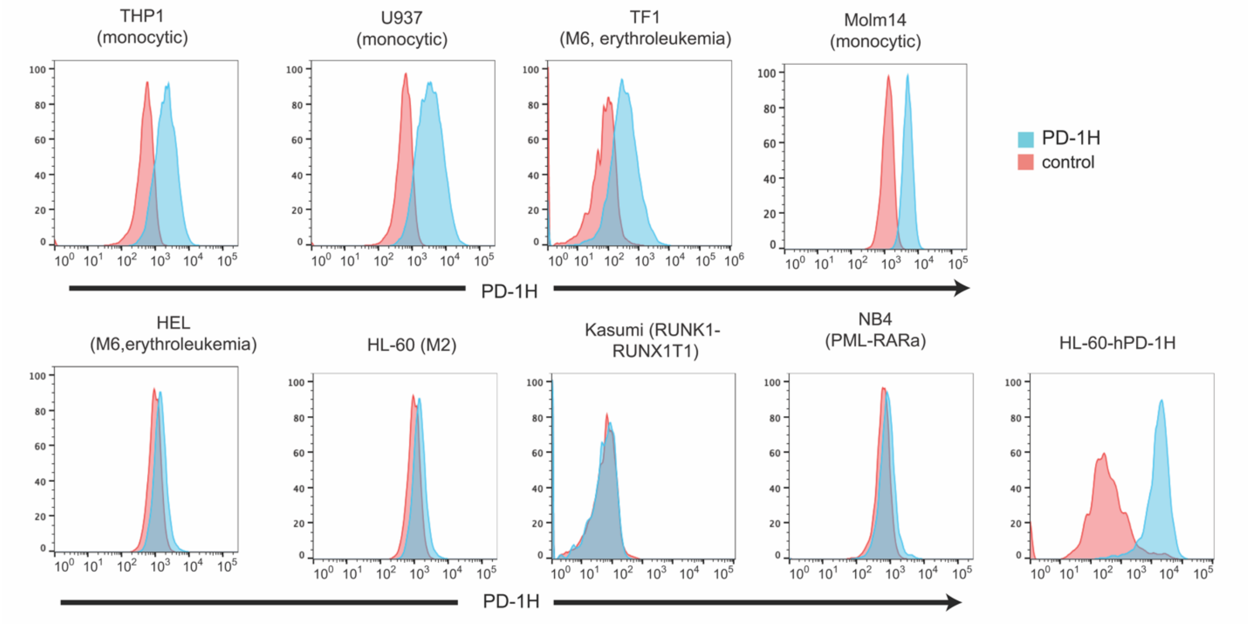

Supplemental Figure 5. Assessment of PD-1H expression in various human AML cell lines using flow cytometry (ΔMFI=1836 (THP1), 3665 (U937), 3000 (TF1), and 3540 (MOLM14) vs. 200 (HEL), 0 (Kasumi1), 515 (HL-60), and 270(NB4)).

Supplemental figure 6

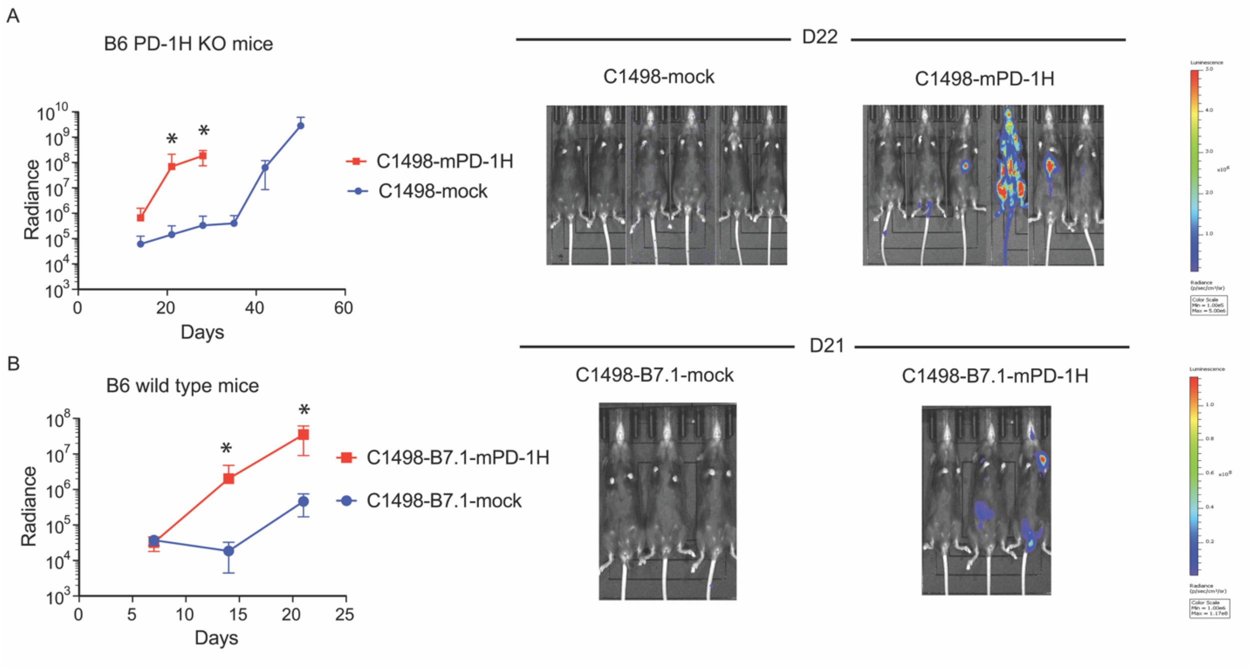

Supplemental Figure 6. In vivo proliferation of C1498FF-mock vs. C1498FF-PD-1H cells in PD-1H KO mice (N=6) (A), C1498FF-B7-1-mock vs. C1498FF-B7-1-PD-1H cells in B6 PD-1H WT mice (N=3) (B). In vivo proliferation was assessed using bioluminescence (representative images on the right side). P value determined by Student's T test at each timepoint. NS: not significant. ***P<0.001, *P<0.05. These experiments were repeated three times. Repeated measures were determined by ANOVA with two factors (P>0.05, no difference among experiments).

Supplemental figure 7

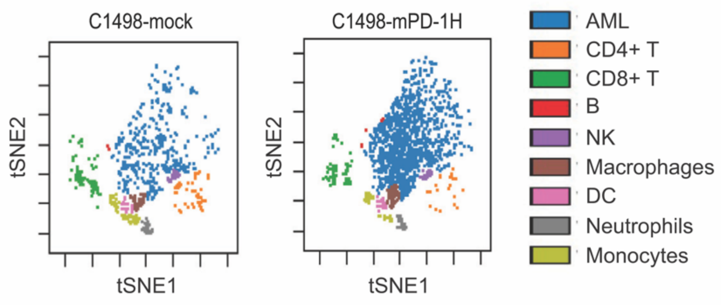

Supplemental Figure 7. Representative viSNE graphs from mass cytometry analysis of C1498FF-PD-1H tumors compared with C1498FF-mock tumors.

Supplemental figure 8

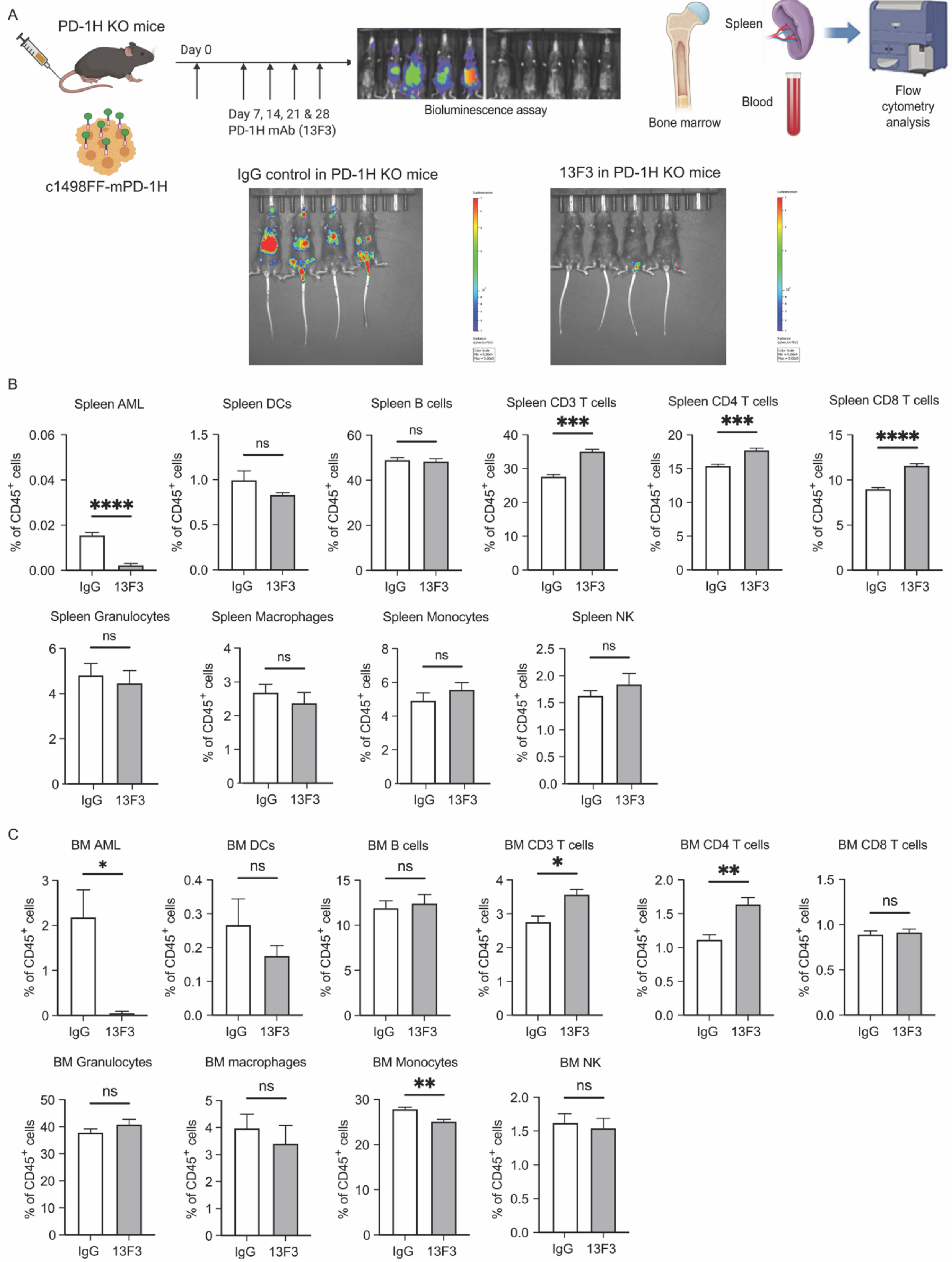

Supplemental Figure 8. Immune microenvironments in hematopoietic tissue of leukemia bearing mice treated with PD-1H blockade.

A. C1498FF-mPD-1H cells (RFP+) were transplanted into PD-1H KO mice. These mice were treated with anti-mPD-1H (13F3) or isotype control. On day 28 post-AML transplant, mice were sacrificed and dissected for getting hematopoietic tissues (B. spleen, C. bone marrow (BM)). The quantity of immune cell subsets (dendritic cells (DCs): CD11c+, B cells: CD19+, T cells: CD3+, CD4+ T cells: CD3+CD4+, CD8+ T cells: CD3+CD8+, granulocytes: CD11b+Ly6G+, macrophages: CD11b+F4/80+, monocytes: CD11b+Ly6C+, natural killer cells (NK cells): NK1.1) was assessed after excluding RFP+ AML cells using multi-color flow cytometry (% CD45+ cells). Antibodies used for flow cytometry was described in table 4. PD-1H blockade increased T cell infiltration in spleen and BM microenvironments of PD-1H+ AML bearing mice.

Supplemental figure 9

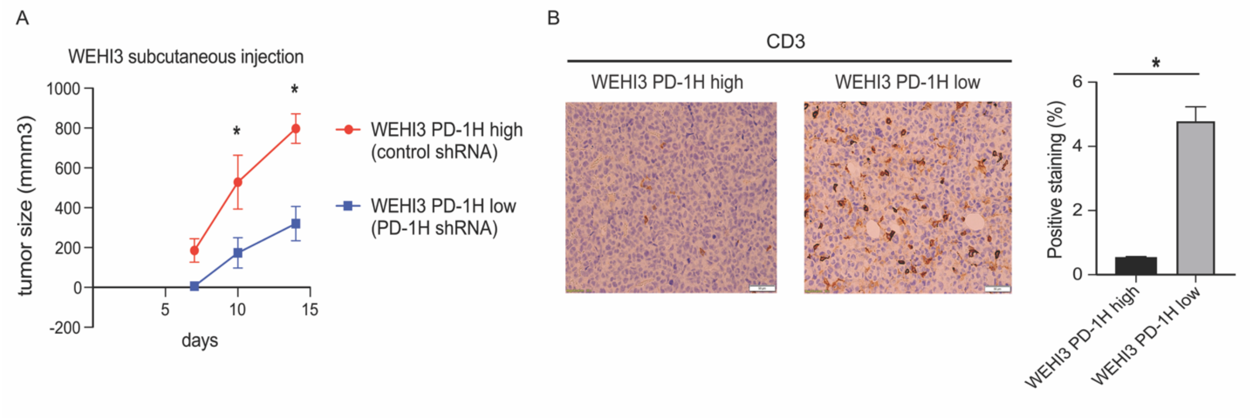

Supplemental Figure 9. Murine AML surface PD-1H (WEHI3) suppresses T cell infiltration in the leukemia tumor environment, leading to immune evasion.

A. Syngeneic mouse model using s.c. injection with WEHI3. WEHI3-PD-1H^{high} cells (scramble shRNA) or WEHI3-PD-1H^{low} cells (PD-1H shRNA) were s.c. injected into the flanks of BALB/c mice, and the tumor volume was assessed (N=5 per group, p<0.05). Mice were sacrificed on day 14 and tumor tissues were removed for IHC.

B. Immunohistochemistry to stain for CD3 in WEHI3-PD-1H^{high} or WEHI3-PD-1H^{low} tumors. CD3^{+} T cells were quantified by ImageJ.

Supplemental figure 10

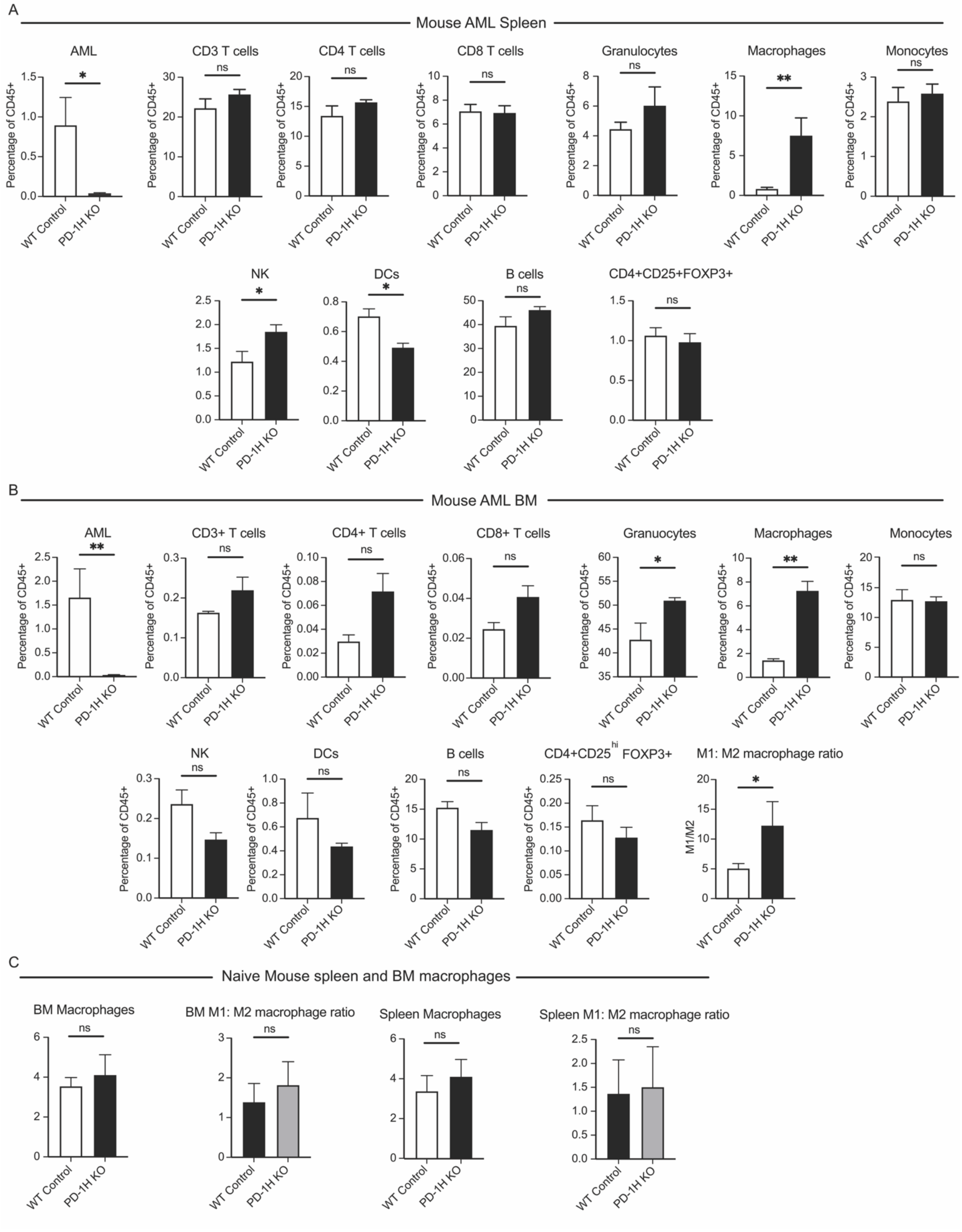

Supplemental Figure 10. Immune microenvironments in hematopoietic tissue of leukemia bearing PD-1H KO or WT mice.

A, B. C1498FF-mock cells (RFP+) were transplanted into PD-1H KO or WT mice. On day 28 post-AML transplant, mice were sacrificed and dissected for getting hematopoietic tissues (A. spleen, B. BM). The quantity of immune cell subsets (dendritic cells (DCs): CD11c+, B cells: CD19+, T cells: CD3+, CD4+ T cells: CD3+CD4+, CD8+ T cells: CD3+CD8+, granulocytes: CD11b+Ly6G+, macrophages: CD11b+F4/80+, M1: CD11b+F4/80+MHC class II+, M2: CD11b+F4/80+CD206+, monocytes: CD11b+Ly6C+, natural killer cells (NK cells): NK1.1, regulatory T cells (Tregs): CD4+CD25^{hi}FoxP3+) was assessed after excluding RFP+ AML cells using multi-color flow cytometry (% CD45+ cells). Antibodies used for flow cytometry was described in table 4. C. The quantity of immune cell subsets, particularly macrophages was assessed in naïve PD-1H KO or WT mice.

Supplemental figure 11

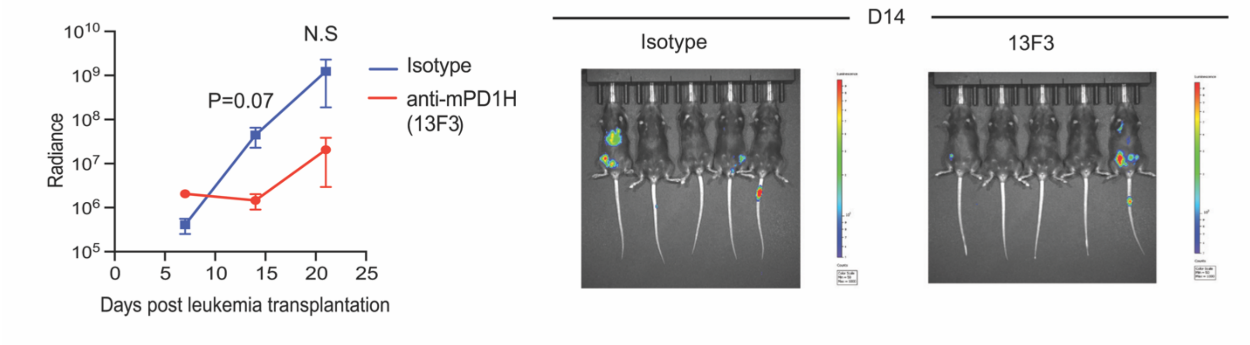

Supplemental Figure 11. In vivo proliferation of C1498FF-mock cells in B6 WT mice following anti-mPD-1H mAb (clone 13F3) treatment. A total of 200 μg of 13F3 or isotype control mAb was intraperitoneally injected every 4 days from day 1 of transplantation of C1498FF-mock cells (total 4 doses). In vivo proliferation was assessed using bioluminescence (representative images on day 14 on the right). Radiance indicates the mean value of 5 mice per group (mean±SED, left side). P value determined by Student's T test at each timepoint. Error bars represent SEM. NS: not significant, *p<0.05.

Supplemental figure 12

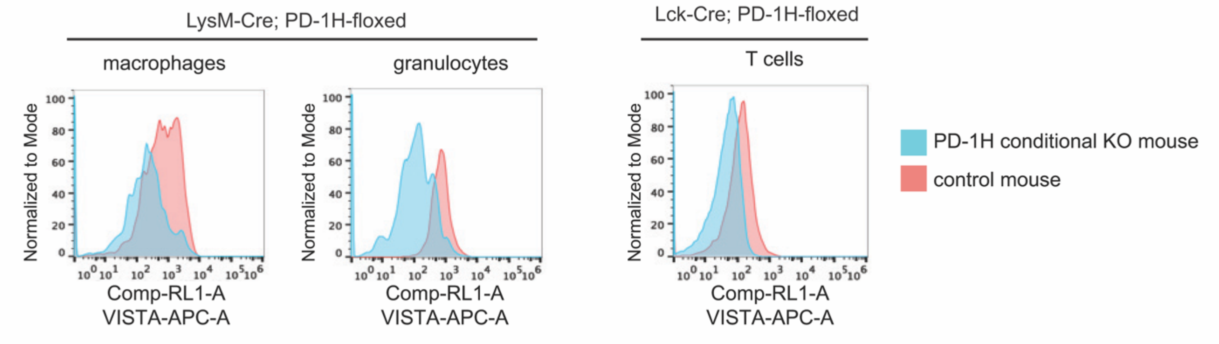

Supplemental Figure 12. PD-1H is deleted in lineage specific knockout mice. Deletion of PD-1H in conditional knockout mice was confirmed by flow cytometry. PD-1H expression was assessed on CD11b+F4/80+ (macrophages) or CD11b+Ly6G+ (granulocytes) population in LysM (lysozyme)-Cre^{+}PD-1H-floxed mice; on CD3+ (T cells) population in Lck-Cre^{+}PD-1H-floxed mice compared with control mice.

Supplemental figure 13

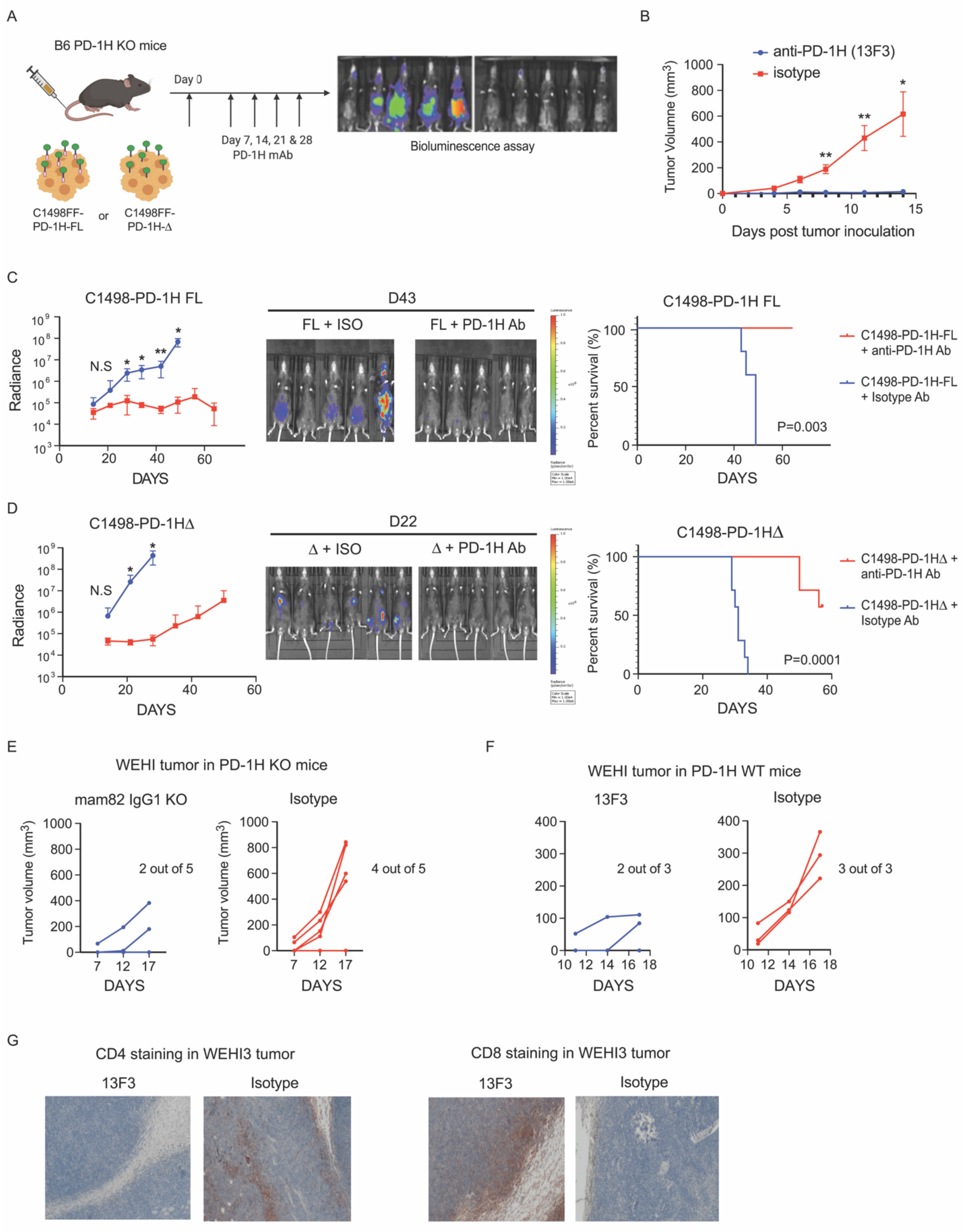

Supplemental Figure 13. Anti-PD-1H mAb reverses the inhibitory effect of AML surface PD-1H on T cells.
A. Syngeneic mouse leukemia model (PD-1H KO mice) using tail vein injection with myeloid leukemia cells expressing full length PD-1H (C1498-PD-1H FL) or PD-1H with an intracellular domain truncation (C1498-PD-1HΔ). B6 PD-1H KO mice were transplanted with C1498-PD-1H FL or C1498-PD-1HΔ. To rule out the possibility that anti-mPD-1H mAb has the effect of antibody dependent cell cytotoxicity (ADCC) and complement dependent cytotoxicity (CDC), we modified the Fc part of the anti-mPD-1H mAb (Baudino et al., 2008). This modified mAb does not bind to the Fc receptor or to complement. Mice were treated with anti-mPD-1H mAb (mam82 IgG1KO) on days 7, 14, 21, and 28 post-transplant. Mice were assessed for in vivo leukemia proliferation using bioluminescence.

B. Blocking effect of anti-mPD-1H mAb (13F3) to reverse the effect of immune evasion induced by mouse AML surface PD-1H in the absence of host derived PD-1H (PD-1H KO mice). In vivo growth of C1498FF-PD-1H s.c. tumor in B6 WT mice following anti-mPD-1H mAb (clone 13F3) treatment was assessed. A total of 200 μg of 13F3 or isotype control mAb was intraperitoneally injected every 4 days from day 0 after s.c. injection of C1498FF-PD-1H cells (total 3 doses). Tumor size on day 8, 11, 14 was significantly smaller in the 13F3 treatment group compared with the isotype treatment group. Mean tumor volume±SED. P value determined by Student's T test at each timepoint. N=4 per group, **p<0.01, *p<0.05.

C, D. Blocking effect of anti-mPD-1H mAb (mam82 IgG1KO) to reverse the effect of immune evasion induced by mouse AML surface PD-1H. Mice were assessed for in vivo leukemia (C1498-PD-1H FL, N=5 per group; C1498-PD-1HΔ, N=6 per group) proliferation using bioluminescence (left) and for survival (Kaplan Meier curve, right). Radiance indicates the mean value per group and error bars represent SEM. P value determined by Student's T test at each timepoint. NS: not significant. *p<0.05, **p<0.01. Experiments were performed twice. Repeated measures were determined by ANOVA with two factors (P>0.05, no difference among experiments).

E. Syngeneic mouse leukemia model using s.c. injection with myeloid leukemia cells expressing PD-1H (WEHI WT). BALB/c PD-1H KO mice were s.c injected with WEHI3 WT cells and treated with anti-mPD-1H mAb (clone mam82 IgG1KO) (on days 0, 5, 10, and 15 post-injection). Tumor size was assessed every 5 days from day 7 post-tumor injection. Only 2 out 5 mice developed tumors in the mam82 IgG1 KO treated group while 4 out 5 mice developed tumors in the isotype control group. Average size of tumors in mam82 IgG1KO treatment group vs. isotype treatment group on day 17: 112 vs. 560 mm^3.

F. Syngeneic mouse leukemia model using s.c. injection with myeloid leukemia cells expressing PD-1H (WEHI WT). BALB/c PD-1H WT mice were s.c injected with WEHI3 WT cells and treated with anti-mPD-1H mAb (clone 13F3) (on days 0, 5, and 10 post-injection). Tumor size was assessed every 5 days from day 10 post-tumor injection. Average size of tumors in 13F3 vs. isotype treatment group on day 17: 65 vs. 293 mm^3. Mice were sacrificed for IHC.

G. IHC to assess T cell infiltration in WEHI3 tumors treated with 13F3 vs. isotype control. 13F3 treatment increased $CD4^+$ and $CD8^+$ T cell infiltration.

Supplemental figure 14

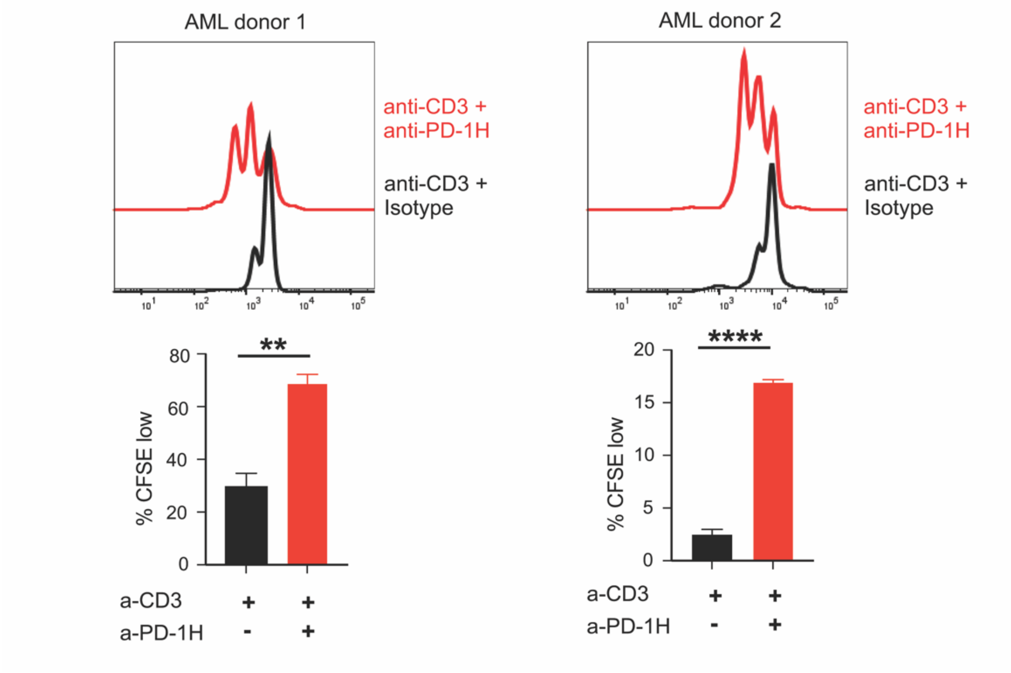

Supplemental Figure 14. Anti-human PD-1H mAb reverses immune evasion induced by human primary AML surface PD-1H.

Following anti-CD3 stimulation in human primary AML BM, in vitro T cell proliferation was assessed by CFSE dilution assay in the presence of anti-human PD-1H mAb. Anti-human PD-1H mAb reverses PD-1H mediated inhibition of T cells in AML BM with PD-1H+ blasts.

Supplemental figure 15

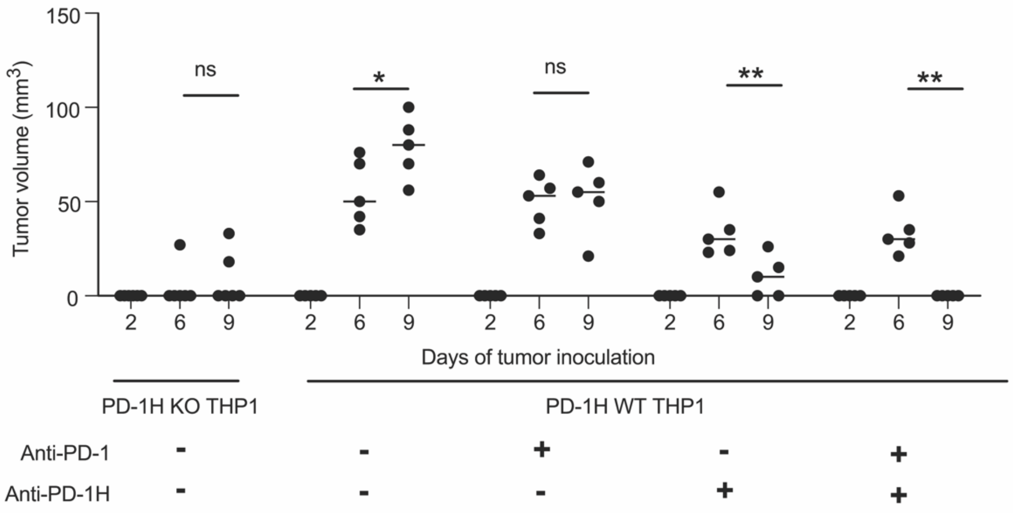

Supplemental Figure 15. A humanized AML mouse model to demonstrate a synergistic anti-leukemia effect of anti-hPD-1 with anti-hPD-1H mAb. Human myeloid leukemia cells expressing PD-1H (THP1-WT) or not expressing PD-1H (THP1-PD-1H KO) were s.c. injected into NSG mice reconstituted with human peripheral blood mononuclear cells. Anti-hPD-1 (100ug) and/or anti-hPD-1H mAb (100ug) were injected on day 7. Tumor volume was assessed on day 2, 6, 9. Mean tumor volume±SED. Error bars represent SEM. N=5. *P<0.05, ***P<0.001, ****P<0.001. P value determined by One way ANOVA.

Supplemental Table 1. List of AML patients for immunohistochemistry

Diagnosis	AML blast PD-1H score	Immune cells PD-1H positive	Immune cells PD-L1 positive	Marrow blast % core	Monocytic differentiation	Karyotype	NGS
AML t(8;21)	1+	Yes, MK, myeloids	Yes, MK, macrophages	40	No	46,XX,t(8;21)(q22;q22)[17]/46,XX[3]	ASXL1 p.Glu635Argfs*15 (VAF not available); PTPN11 pGly60Arg (VAF not available)
AML t(8;21)	0	Yes, MK, rare myeloids	Yes, MK, macrophages	60	No	45,X,-X,t(8;21)(q22;q22)[15]/46,XX[5]	Normal
AML t(8;21)	1+	Yes, MK, myeloids	Yes, MK, macrophages	20	No	46,XX,t(8;21)(q22;q22)[14]/46,XX[6]	KRAS p.Gly12Asp (11%)
AML inv(16)	1+	Yes, MK, myeloids	Yes, MK	70	Yes	46,XY,inv(16)(p13.1q22)[17]/46,XY[3]	KRAS p.Gln61His (VAF not available)
AML inv(16)	2+	Yes, MK, ?myeloids	Yes, MK, macrophages	60	Yes	46,XY,inv(16)(p13.1q22)[15]/46, XY[5]	FLT3-ITD p.Gln580_Val581ins4 (3%); NRAS p.Gly12Asp (5%)
AML inv(16)	2+	Yes, MK, myeloids	Yes, MK, macrophages	60	Yes	46,XY,inv(16)(p13.1q22)[19]/46, XY[1]	NRAS p.Gln61Arg (37%); FLT3 p.Asp835Tyr (4%); FLT3 p.Asp835Pro (2%); VUS in WT1 p.Arg158His (50%)
APL	1+	Yes, MK	Yes, MK, rare macrophages	70	No	46,XX,t(15;17)(q24;q21)[17]/46, XX[3]	N/A
APL	1+	Yes, MK	Yes, MK, macrophages	80	No	46,XY,t(15;17)(q24;q21)[11]/46, XY[9]	N/A
APL	2+	Yes, MK	Yes, MK	70	No	46,XY,t(15;17)(q24;q21)[15]/46,XY[5]	N/A
Complex karyotype	2+	Yes, MK	Yes, MK	90	Yes	56~57,XY,+Y,+6,+8,+8, t(9;11)(p22;q23), +11, add(12)(p11.2), +14,+18,+1	U2AF1 p.Ser34Phe (6%)

						8,+19,+21, +22[cp19]/46,XY[1]	
Complex karyotype	3+	Yes, MK, ?myeloids	Yes, MK, macrophages?	30	No	45,XY,+1,+add(1)(p13),psu dic(1;1)(q21;q21),-2, add(2)(p11.2),-7,del(9)(q13q22),der(12)del(12)(p13)add(12)(q13),add(13)(q22), add(17)(p11.2),-20,+mar[cp7]/46,XY[13]	TP53 p.Arg342* (57%); BCOR p.Ser423Phefs*16 (25%); VUS in BCORL1 (6%)
Complex karyotype	2+	Yes, rare MK	Yes, MK, macrophages	70	No	45,XY,?inv(17)(p13q21),-20,del(21)(q22)[11]/45,XY,del(6)(p21),add(11)(p15),-17, del(21)(q22)[7]/45,XY,del(4)(q25),-17[2]	BCOR p.Gln1208Thrfs*8 (89%); TP53 p.Arg306* (86%)
Monosomal	3+	Y	Yes, rare cells	50	No	45,XX,add(1)(q21),-5,add(6)(q25),+8,-13,-15,-17,-17,?del(21)(q22), +3mar[6]/46,XX[14]	TP53 p.Gly244Asp (52%)
Monosomal	1+	Y, MK, myeloids	Yes, rare plasma cells	25	No	43~44,XX,del(3)(q21),add(7)(p21), ?i(9)(q10),-9,-9,-18,add(18)(q23), add(21)(p11.2)[cp18]/46,XX[2]	TP53 p.His179Arg (25%)
Monosomal	1+	Y, MK, myeloids	Yes, MK	60	No	43~47,XX,del(5)(q13q33),-7,+8,add(8)(q24),-10,-11,add(12)(p13), add(13)(p11.2),-14,-16,-	TP53 p.Leu111Pro (72%); VUS in ZRSR2 p.Ser447_Arg448insGlnSer (46%)

						22,+r,+1~4 mar[cp19]/46,XX[1]	
AML	1+	Yes, MK, PC (rare cells)	Yes, PC (rare cells)	90	No	46,XX[20]	GATA2 p.Ala318Thr (13%)
AML	0	Yes, MK, rare myeloids	Yes, MK, rare macrophages	90	No	47,XX,+13[16]/48,idem,+13[1]/46,XX[3]	ASXL1 p.Glu635Argfs*15 (40%); BCOR p.Glu1076Glyfs*3 (37%); RUNX1 p.Ser388 (85%); SRSF2 p.Pro95His (48%)
AML	3+	Yes, MK	Yes, MK, rare macrophages	90	No	46,XY,del(7)(q22)[14]/46,XY[6]	FLT3-ITD p.? (1%); FLT3-ITD p.Lys602_Trp603ins6 (<1%); IDH1 p.Arg132His (5%); NRAS p.Gly12Asp (4%); PTPN11 p.Glu76Ala (35%); RUNX1 p.Gln415Profs*185 (46%); U2AF1 p.Ser34Phe (44%); VUS in BCOR p.Arg1375Trp (91%)
Monocytic AML	3+	Yes, MK, ?myeloids	Yes, MK	50	Yes	47,XX,+8[4]/46,XX[16]	DNMT3A p.? (45.2%); NPM1 p.Trp288Cysfs*12 (34.7%); FLT3 p.Asp835Tyr (34.7%); FLT3-ITD p.Gly583_Tyr599dup (1.3%); VUS in ASXL1 p.Lys1532* (VAF not available)
Monocytic AML	3+	No/NA	No/NA	90	Yes	46,XX[20]	NPM1 p.Trp288Cysfs*12 (27.7%); TET2 p.Gln321* (31.9%);

							TET2 p.Lys1493Serfs*78 (31.8%)
Monocytic AML	3+	Yes, MK	Yes, MK, rare macrophages	90	Yes	46,XY[20]	FLT3 p.Asp835Tyr (41%); NPM1 p.Trp288Cysfs*12 (47%)

AML, acute myeloid leukemia; APL, acute promyelocytic leukemia; MK, megakaryocyte; N/A, not applicable; NGS, next generation sequencing; PC, plasma cell; VAF, variant allele frequency

Supplemental Table 2. List of AML patients for flow cytometry

Diagnosis	Phenotype	Cytogenetics	PD-1H delta MFI
ACUTE MYELOID LEUKEMIA WITH MYELODYSPLASIA-RELATED CHANGES/Monocytic Feature	CD14+ CD64+ CD11b+ CD33+ CD13variable+ CD16subset+ CD34- CD117- with aberrant CD56 positivity and HLADr negativity, most consistent with monoblasts/promonocytes.	49,XY,+4,+8,+21[3]/46 ,XY[12]	12374
ACUTE MYELOID LEUKEMIA INVOLVING 30% OF TOTAL CELLULARITY	CD45dim+ CD13+ CD34+ CD7dim- HLA-DR+ CD33+ MPO+, CD117var+	46,XY,del(9)(p21)[3]/4 5,idem, -Y[12]	1851
ACUTE MYELOID LUKEMIA INVOLVING 20% OF BONE MARROW CELLULARITY	CD34+ CD117+ HLADRdim+ CD13+ CD33var+ CD11b- CD7- CD64- CD14- CD10-	47,XX,del(5)(q13q34), +8[17] (del5 q and tri 8 in a subclone)	424
PERSISTENT MYELOID NEOPLASM WITH 13% BLASTS (Erythroid dysplasia)	CD45dim+ CD13dim+ CD34+ CD117+ CD33+ HLADR+ MPO+ myeloblasts with very dim, aberrant CD7 expression.	XY[20]	695
ACUTE MYELOID LEUKEMIA, 40% BLAST	CD45dim+ CD13+ CD34+ CD117+ CD33+ HLADR+ CD38+ MPO+ TdT-. Blasts also demonstrate dim, aberrant CD7 expression.	46,XY,del(9)(q21q32)[5]/46, XY[10]	675
ACUTE MYELOID LEUKEMIA (90% BLASTS)	CD45dim+ CD34dim+ TdT- MPO++ CD117dim+ CD13+ CD33+ HLADRdim+ CD14- CD64- CD56- CD7-	46, XX[20]	1259
CMML-->AMML	CD38++ CD15- CD56- CD19- CD2- CD7-. (Marked increased monocytes with approximately 28-30% of total marrow cellularity consists of CD64+ monocytes with variable CD14 expression and dim expression of CD56. There are normal numbers of myeloblasts with normal myeloid scatter by CD45/SSC. There is an abnormal CD10/CD13/CD16/CD11b myeloid maturation pattern suggesting myeloid dysmaturation)	46,XX,del(4)(q21.23q2 5)[7]46,idem,r(10)(p11. 2q23)[3]/46,idem, der(10)t(3;10)(q21;p11 .2)[3]/ /47,idem,+8, der(13)t(3;13)(q21;p11 .2)[cp2]/46,idem, der(22)t(3;22)(q21;q13)[1]	15320
ACUTE MYELOID LEUKEMIA INVOLVING 30-40% OF TOTAL BONE MARROW CELLULARITY	CD34+ CD117subset+ CD33- CD7- CD13+ CD11b- CD16- CD2- CD10- CD19- CD5- HLADR+ CD64- glycophorin- CD41a- TDT var+ CD79a-, MPO+ CD45dim+	46,XY, t(9;22)(q34;q11.2)[15]	963
Acute monocytic leukemia (AML M5)	CD34+ CD117- CD33+ HLA DR+ MPO- CD13+ (subset) CD11B+ CD64+	46,XY[20]	25000
Acute myelomonocytic leukemia (AML M4)	CD45dim+ CD34- CD13+ CD33+ HLADR+ CD117+ CD7- CD11b dim/- CD16- CD64dim+.	46,XX[18]	3122
Acute myelomonocytic leukemia (AML M4)	About 15-17% of total circulating cells are CD45dim+ CD13+ CD34+ CD117+ CD33dim+ HLA-DR+ MPO- myeloblasts. In addition, about 30% of total cells are monocytes by immunophenotype (CD45dim+ CD13+ CD11bdim+ HLA-DRdim+ CD33+ CD14dim+ CD16dim+ CD64+ CD4dim+ CD34- CD117- MPO-)	45,Y,der(X)t(X;7)(q22; q22),t(1;12)(q31;q21), del(3)(p21p23),add(3)(q21),der(4)t(4;15)(q21; q15), del(5)(q15),- 7,del(15)(q15)[cp14]/4 6, XY[1]	2840

Acute myelomonocytic leukemia (AML M4)	CD33+ CD13dim+ CD64dim+ CD56- CD38+ CD14- MPOsubset+ CD117- CD34- HLADR+ CD11bdim MPO subset+	48,XX,+6,t(11;17)(q23;q25),+13[15]/50,idem,+4,+8[3]/46, XX[2] (KMT2A gene rearrangement)	243
AML	CD45dim+ CD34dim/- CD33+ CD117var+ CD7- HLADRvar+ CD56- CD14- CD64dim+ CD38var+ MPO+.	XX[20]	601
AML with monocytic differentiation	CD117(subset)+ CD34- CD33+ HLADR- CD13+ CD14- CD4- CD64- CD11b- CD19dim+ CD10- TdT- CD79A- MPO(variable) CD3- CD45dim+	46, XX[20]	21103
Poorly differentiated acute myeloid leukemia	CD45dim+ CD13+ CD34+ CD117+ HLADR+ CD33dim+ CD11b- CD16- CD19- CD10- CD7- CD56- CD3- CD64- MPOdim/- TdT- CD79a-	46,XX,del(1)(p13p31)[6]/46,XX[9]	260
AML with monocytic differentiation	CD34+ CD117+ TdT- CD45- CD13+ MPO(var)+ CD7(dim)+ CD19- CD79a- CD5- CD8- CD10- CD16- CD56- CD11b- cytoCD3- HLADR+ CD33(dim)+. A subset of cells are CD4(dim)+ CD14+ CD45dim)+ SSClow	46,XY,del(21)(q21q22)[9]/46, XY[12] (21q deletion does not include the RUNX1 gene at 21q22.12)	917
Relapsed AML-myelomonocytic	70% of total cells with a CD45dim+, CD34+, CD117dim+, HLADRdim/-, CD7-, CD13+, CD19-, MPO+, TdT-, CD33+ myeloblast immunophenotype. Approximately 22% of cells are CD45dim+, CD13+, CD34-, CD117dim+, CD33+, HLADRdim/-, CD64dim+ blasts likely reflecting an immature monocyte phenotype.	46,XX, t(13;16)(q22;p13.3)[20]	10367
AML with monocytic differentiation	64% of total cells are variable side scatter, CD45dim+ CD34- CD117- CD33+ CD64dim+ CD4+ CD14- CD56+ CD11bvar+ CD13- MPO++ CD16- Glyco- CD41a- (negative for all T- and B-cell markers) myeloblasts with some evidence of monocytic differentiation	50,XX,+i(5)(p10)x3,+8[8]/46, XX[7]	25088
AML t(8;21)	low SSC/CD45dim+ blasts, which are CD34+ CD117+ HLADR+ CD33dim+ CD13+ CD7- CD19dim+ MPOdim+ CD11b- CD64dim+ and CD4subset[dim]+. The blasts do not label for any other B or T cell markers.	46,XY, t(8;21)(q21.3;q22)[15]	944
AML t(8;21)	CD45dim+ CD34+ HLA-DR+ CD117+ CD33+ CD13+ MPO+ CD7- CD16- CD11b- glycophorin A- CD41a-, CD56subset+ CD64dim/- CD14- TdT-	46,XX, t(8;21)(q21.3;q22)[20]	323
AML t(15;17)	CD13+ CD33++ CD117+ CD34- HLA-DR- CD64+ CD11b- CD16-.	46,XY,t(15;17)(q24.1;q21.2)[14]/46, XY[1]	1968
AML t(8;21)	High SSC CD34+ CD16/56+ CD117variable+ HLA-DR+ CD13variable+ MPO+ CD33- CD11b- CD2dim- CD64- CD14- CD19- CD20- CD3- CD7- CD5- CD10- CD41a- glycophorin a- TdT- CD79a-	46,XX,t(8;8;21)(q22;p21;q22)[10]/47,idem,+4[3]/46, XX[3] ish t(8;8;21)(q22;p21;q22)(RUNX1T1+, RUNX1+;RUNX1T1+; RUNX1+)	338
AML t(8;21)	MPO++ CD34(variable)+ CD117(variable)+ TdT- CD79a- HLADR+ CD13+ CD33- CD2- CD16- CD10- CD19- CD56dim+ CD14- CD64- CD41a- Glyco-	46,XX, t(8;21)(q22;q22)[15]	601
AMML	CD45dim+ CD33+ CD14+ HLADR+ CD13+ CD4dim+ CD11b+ CD16- CD64+ CD56+ CD8- MPO+ with a small population of CD34+ CD117+ CD33+ HLADR+ myeloblasts (2.5%).	46, XY[21]	28227

AML (inversion 16)	Approximately 25% of cells are immature myeloid elements by CD13/SSc criteria. About 7-8% of cells are CD13+ CD34+ myeloblasts and the remaining cells are CD64+ CD14variable+ monocytes. There is also evidence of myeloid dysmaturation by CD10/CD13/CD16/CD11b pattern.	46,XY, inv(16)(p13.1q22)[15] Abnormal clone detected.	491

AML, acute myeloid leukemia; AMML, acute myelomonocytic leukemia; CMML, chronic myelomonocytic leukemia; MFI, mean fluorescence intensity

Supplemental table 3. Antibody list for mass cytometry for mouse immune phenotyping

Target	Clone	Vendor	Cat#:
CD45	30-F11	Fluidigm/Standard BioTools	3089005B
Beads			
PDCA-1	927	BioXcell	BE0311
CD11c	N418	Fluidigm/Standard BioTools	3142003B
CD69	H1.2F3	Fluidigm/Standard BioTools	3143004B
B220	RA3-6B2	Fluidigm/Standard BioTools	3144011B
CD4	RM4-5	Fluidigm/Standard BioTools	3145002B
F4/80	BM8	Fluidigm/Standard BioTools	3146008B
CD68	FA-11	Biolegend	137002
FAS	SA367H8	Biolegend	152608
CD19	6D5	Fluidigm/Standard BioTools	3149002B
CD25	PC61	Biolegend	102002
Ly-6G	1A8	Fluidigm/Standard BioTools	3151010B
CD3e	145-2C11	Fluidigm/Standard BioTools	3152004B
CD8a	53-6.7	Fluidigm/Standard BioTools	3153012B
PD-L1	10B5	Lab own	
Foxp3	FJK-16s	Fluidigm/Standard BioTools	3158003A
PD-1	29F.1A12	Fluidigm/Standard BioTools	3159024B
TBET	4B10	Fluidigm/Standard BioTools	3161014B
CD80	16-10A1	Biolegend	104747
Ly-6C	HK1.4	Fluidigm/Standard BioTools	3162014B
CXCR3	CXCR3-173	Biolegend	126502
CD62L	MEL-14	Fluidigm/Standard BioTools	3164003B
4-1BB	3H3	Lab own	
CCR6	29-2L17	Biolegend	129802
Ki-67	B56	Fluidigm/Standard BioTools	3168022D
NK1.1	PK136	Fluidigm/Standard BioTools	3170002B
CD44	IM7	Fluidigm/Standard BioTools	3171003B
CD11b	M1/70	Fluidigm/Standard BioTools	3172012B
GZMB	GB11	Fluidigm/Standard BioTools	3173006B
LAG-3	C9B7W	Fluidigm/Standard BioTools	3174019B
CD127	A7R34	Fluidigm/Standard BioTools	3175006B
EOMES	Dan11mag	Invitrogen/ThermoFisher	14-4875-82
Cell-ID™ Intercalator-Ir—125 µM		Fluidigm/Standard BioTools	201192A
Cell-ID™ Intercalator-Ir—125 µM		Fluidigm/Standard BioTools	201192A
Cell-ID™ Cisplatin-195Pt		Fluidigm/Standard BioTools	201195
MHC class II	M5/114.15.2	Fluidigm/Standard BioTools	3209006B

Supplemental table 4. Antibody list for flow cytometry for mouse immune phenotyping

Item	Vendor	Cat. #
APC/Cyanine7 anti-mouse CD45 Antibody	Biolegend	103116
PerCP/Cyanine5.5 anti-mouse CD3 Antibody	Biolegend	100218
Alexa Fluor® 700 anti-mouse CD8a Antibody	Biolegend	100730
Alexa Fluor® 488 anti-mouse NK-1.1 Antibody	Biolegend	108718
Brilliant Violet 421™ anti-mouse CD4 Antibody	Biolegend	100438
PE/Cyanine7 anti-mouse Ly-6G Antibody	Biolegend	127618
APC anti-mouse F4/80 Antibody	Biolegend	123116
Brilliant Violet 570™ anti-mouse CD11c Antibody	Biolegend	117331
PE Rat Anti-CD11b	BD	557397
Brilliant Violet 785™ anti-mouse Ly-6C Antibody	Biolegend	128041
Brilliant Violet 650™ anti-mouse I-A/I-E Antibody	Biolegend	107641
PE/Cyanine5 anti-mouse CD69 Antibody	Biolegend	104510
CD206 (MMR) Monoclonal Antibody (MR6F3), PE-eFluor 610	Thermo Fisher	61-2061-82
Brilliant Violet 605™ anti-mouse CD19 Antibody	Biolegend	115540
